# DWI-based radiomic signature: potential role for individualized adjuvant chemotherapy in intrahepatic cholangiocarcinoma after partial hepatectomy

**DOI:** 10.1186/s13244-022-01179-7

**Published:** 2022-03-04

**Authors:** Yang Yang, Xianlun Zou, Wei Zhou, Guanjie Yuan, Daoyu Hu, Yaqi Shen, Qingguo Xie, Qingpeng Zhang, Dong Kuang, Xuemei Hu, Zhen Li

**Affiliations:** 1 Department of Radiology, Tongji Hospital, Tongji Medical College, Huazhong University of Science and Technology, 1095 Jiefang Avenue, Qiaokou District, Wuhan, 430030 Hubei China; 2 Department of Biomedical Engineering, Huazhong University of Science and Technology, 430074, Wuhan, Hubei China; 3 School of Data Science, City University of Hong Kong, Kowloon, Hong Kong China; 4 Department of Pathology, Tongji Hospital, Tongji Medical College, Huazhong University of Science and Technology, 1095 Jiefang Avenue, Qiaokou District, Wuhan, 430030 Hubei China

**Keywords:** Individualized therapy, Intrahepatic cholangiocarcinoma, Prognosis, Radiomics

## Abstract

**Objectives:**

To develop a diffusion-weighted imaging (DWI) based radiomic signature for predicting early recurrence (ER) (i.e., recurrence within 1 year after surgery), and to explore the potential value for individualized adjuvant chemotherapy.

**Methods:**

A total of 124 patients with intrahepatic cholangiocarcinoma (ICC) were randomly divided into the training (*n* = 87) and the validation set (*n* = 37). Radiomic signature was built using radiomic features extracted from DWI with random forest. An integrated radiomic nomogram was constructed with multivariate logistic regression analysis to demonstrate the incremental value of the radiomic signature beyond clinicopathological-radiographic factors. A clinicopathological-radiographic (CPR) model was constructed as a reference.

**Results:**

The radiomic signature showed a comparable discrimination performance for predicting ER to CPR model in the validation set (AUC, 0.753 vs. 0.621, *p* = 0.274). Integrating the radiomic signature with clinicopathological-radiographic factors further improved prediction performance compared with CPR model, with an AUC of 0.821 (95%CI 0.684–0.959) in the validation set (*p* = 0.01). The radiomic signature succeeded to stratify patients into distinct survival outcomes according to their risk index of ER, and remained an independent prognostic factor in multivariable analysis (disease-free survival (DFS), *p* < 0.0001; overall survival (OS), *p* = 0.029). Furthermore, adjuvant chemotherapy improved prognosis in high-risk patients defined by the radiomic signature (DFS, *p* = 0.029; OS, *p* = 0.088) and defined by the nomogram (DFS, *p* = 0.031; OS, *p* = 0.023), whereas poor chemotherapy efficacy was detected in low-risk patients.

**Conclusions:**

The preoperative DWI-based radiomic signature could improve prognostic prediction and help to identify ICC patients who may benefit from postoperative adjuvant chemotherapy.

**Supplementary Information:**

The online version contains supplementary material available at 10.1186/s13244-022-01179-7.

## Key points

- The DWI-based radiomic signature could predict early recurrence in patients with ICC.
- The radiomic signature could stratify ICC patients into distinct survival outcomes.
- High-risk patients defined by radiomic signature could benefit from chemotherapy.
- The radiomic signature showed incremental value beyond routine prognostic factors.

## Introduction

Intrahepatic cholangiocarcinoma (ICC) is the second most frequent form of primary hepatic malignant tumor and accounts for up to 5–10% of primary liver cancers [1]. In recent years, the incidence of ICC is increasing worldwide [2, 3]. Partial hepatectomy is the only potentially curative therapy for ICC [4, 5]. However, the 5-year survival rate after surgery is only 21–35% [6, 7]. The main reason for the unfavorable outcome is the high incidence of recurrence [8]. Approximately 37.9–60% of patients occur local recurrence and/or distant metastases within 1 year after resection, with a median overall survival (OS) as low as 16.3 months [9, 10].

To reduce the risk of recurrence and improve survival, postoperative adjuvant chemotherapy was recommended by clinical practice guidelines for patients with resected ICC [11, 12]. Adjuvant chemotherapy reduced the risk of relapse by 17% and gained a mean OS benefit of 4 months in an unselected population [13, 14]. However, it should be pointed out that not all patients could benefit from additional adjuvant chemotherapy, and particularly, some may even be harmed by chemotherapy-related toxicity [15, 16]. Although a few studies suggest that patients with features such as positive resection margins are more likely to benefit from adjuvant chemotherapy, this feature is far from adequate for patient selection [13, 17]. Thus, identifying novel and powerful biomarkers for selecting patients who may benefit from adjuvant chemotherapy is urgently needed.

Radiomics, which converts medical images into numerous quantitative features and provides important information on the entire underlying intra-tumor heterogeneity and cancer phenotype, is a non-invasive and easily accessible approach to develop cancer biomarkers [18]. Contrast-enhanced magnetic resonance imaging (MRI) based radiomics has been successfully used to predict the status of microvascular invasion and the recurrence of ICC [19–21]. However, there were still few radiomics studies focused on the prediction of adjuvant chemotherapy benefits for patients with ICC.

Furthermore, diffusion-weighted imaging (DWI) which based upon measuring the random Brownian motion of water molecules, is a routine modality for cancer imaging [22]. DWI and the corresponding apparent diffusion coefficient (ADC) maps could reflect cellular density and architectural change, and have been proved to be of great importance in evaluating the biological behavior and predicting the prognosis of ICC [23, 24]. The clinical utility of DWI-based radiomic signature for ICC patients has not been explored.

In this study, we developed a preoperative DWI-based radiomic signature for predicting early recurrence in ICC patients who underwent partial hepatectomy. Further, we explored the potential value of the radiomic signature and the radiomic nomogram for selecting patients who may benefit from adjuvant chemotherapy.

## Materials and methods

This study was approved by the Institutional Review Board at our institution and the requirements for informed consent were waived owing to its retrospective nature.

### Patients

**Fig. 1:**
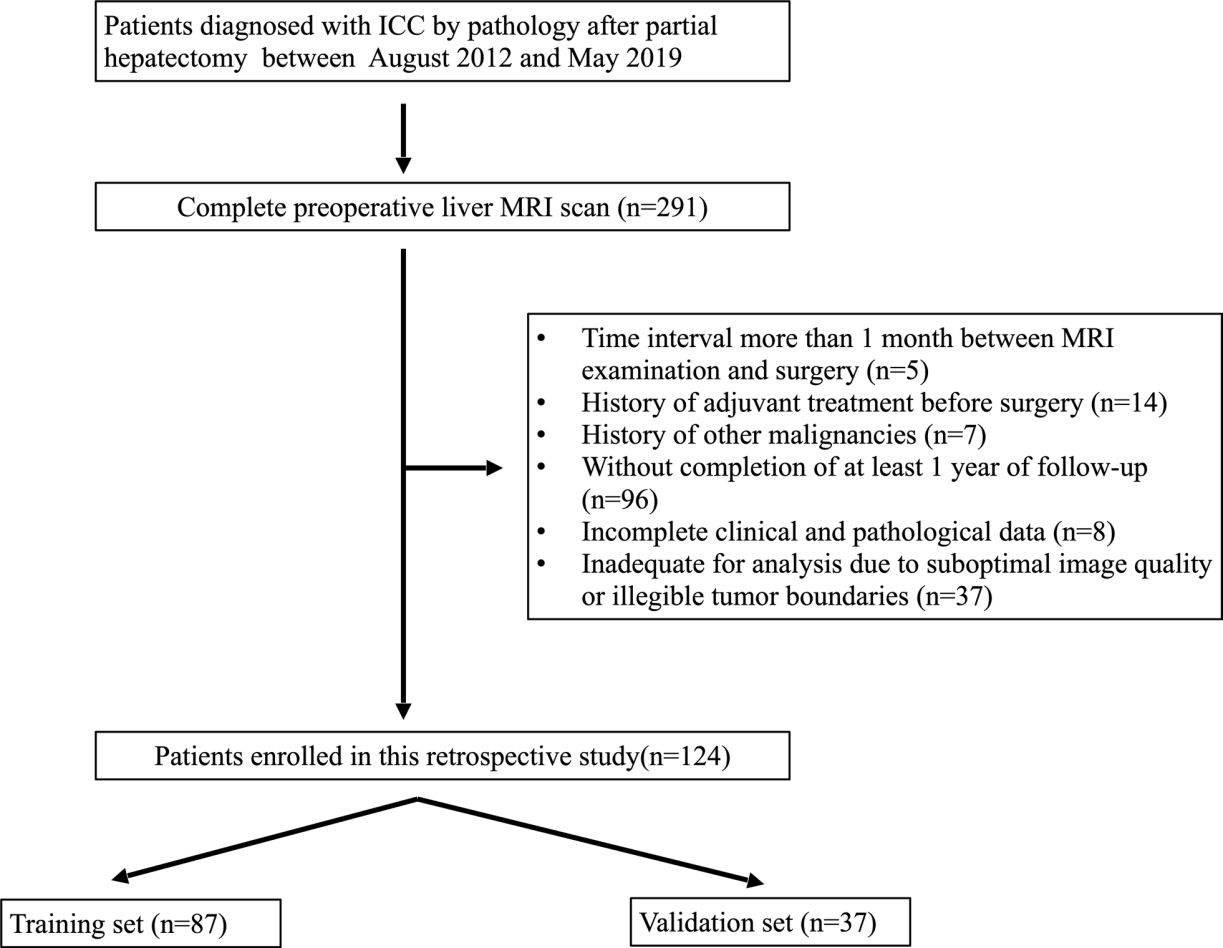
Flow chart of inclusion and exclusion criteria

From August 2012 to May 2019, consecutive patients who underwent liver MRI scans preoperatively and were diagnosed with ICC by pathology after partial hepatectomy in our institution were included. The exclusion criteria for our study were as follows: (a) patients who underwent preoperative MRI scan more than 1 month before surgery (*n* = 5); (b) patients with a history of adjuvant treatment before surgery (*n* = 14); (c) patients with a history of other malignancies(*n* = 7); (d) patients without completion of at least 1 year of follow-up (*n* = 96); (e) patients with incomplete clinical and pathological data (*n* = 8); (f) inadequate for analysis due to suboptimal image quality or illegible tumor boundaries (*n* = 37). The inclusion, exclusion criteria and recruitment pathway of patients was shown in Fig. 1. A total of 124 patients were randomly assigned to the training set (*n* = 87) and the validation set (*n* = 37) in a ratio of 7:3. Clinical characteristics, laboratory examination results were reviewed retrospectively from electronic medical records. Laboratory data including alpha-fetoprotein (AFP), carcinoembryonic antigen (CEA), and carbohydrate antigen 19-9 (CA19-9) were acquired within 2 weeks before surgery. The histopathologic parameters included the presence of macrovascular and microvascular invasion, histologic differentiation, lymph node metastasis, tumor, node, and metastasis (TNM) staging, and surgical margin status. TNM staging was determined based on the eighth edition of the American Joint Committee on Cancer (AJCC) staging system [25].

Postoperative adjuvant chemotherapy regimen included capecitabine alone, gemcitabine combined with capecitabine, or gemcitabine combined with cisplatin for 4–6 courses.

### MR imaging protocol

MR imaging was performed by using a 3.0-T or 1.5-T system. The scanning protocol included MR plain scan, DWI, and contrast-enhanced images. ADC maps were calculated automatically voxel by voxel using a monoexponential function with b values of 0 and 1000 s/mm^2^. Detailed information on MRI protocol was listed in Additional file 1: Table S1.

### MR radiographic feature analysis

Two abdominal radiologists (Y.Y. and H.X.M., with 7 and 12 years of experience, respectively) evaluated the imaging features independently. Interobserver agreement was evaluated. Any disagreement was resolved through consultation with a third abdominal radiologist (H.D.Y. with 38 years of experience). The largest lesion was used for evaluation of the imaging features when a tumor contained multiple lesions. (a) The multifocal tumor was defined as tumors with more than one focus, including intrahepatic metastases and satellite nodules. (b) Tumor diameter was measured as the maximum dimension of the tumor on the axial plane for which the tumor had the largest cross-sectional diameter. In this study, the mean tumor diameter measured by two radiologists was used for follow-up analysis. (c) Irregular tumor margin was considered when a tumor with nonsmooth margins or had a budding portion at its periphery protruding into the liver parenchyma [26]. (d) Arterial enhancement patterns (diffuse hyperenhancement, hyperenhanced portion of the tumor in more than 70% of the tumor area; peripheral rim enhancement, irregular ring-like enhancement in 10–70% of the tumor area with relatively hypoenhancement central areas; diffuse hypoenhancement [27]). (e) Enhancement pattern (wash-out pattern, arterial enhancement with washout on portal or delayed phase; persistent enhancement, hyperenhancement in arterial phase and persistent enhancement in portal and delayed phase; gradual enhancement; and no or minimal enhancement) [27, 28]. (f) Peritumoral enhancement in the arterial phase, as grossly hyperenhancement outside the tumor border, and became isointense in later dynamic phases compared with the background liver parenchyma [29]. (g) Peritumoral biliary dilatation. (h) Target sign on diffusion-weighted images, a peripheral ring-like hyperintense with central hypointense areas [30].

### MR radiomic feature analysis

#### Region-of-interest segmentation and radiomic feature extraction

Region of interest (ROI) on DWI (*b* = 1000 s/mm^2^) including the whole volumes of the tumor was manually segmented by one abdominal radiologist (read1, Y.Y. with 7 years of experience) using an open-source imaging platform (3D Slicer, version 4.11; http://www.slicer.org), then the ROI was copied to corresponding ADC maps. When the tumor lesions were not shown clearly on high b value DWI, DWI (*b* = 0 s/mm^2^) and contrast-enhanced images were used as references to improve the accuracy of segmentation after image registration. After 4 weeks, 20 patients were randomly selected and their ROIs were segmented again by two abdominal radiologists (read1, Y.Y., and read2, Z.X.L with 7 and 4 years of experience, respectively) to evaluate intra- and inter-observer repeatability.

Image preprocessing and feature extraction were performed using PyRadiomics (version 3.0.1; Computational Imaging and Bioinformatics Lab, Harvard Medical School), which were compatible with the Image Biomarker Standardization Initiative (IBSI) [31]. Voxels in each ADC map (*b* = 1000 s/mm^2^) were resampled to isotropic 1 × 1 × 1 mm^3^ [31–33]. MRI signal intensity normalization and gray value discretization (bin width of 16) were performed before the feature extraction. Radiomic feature classes consisted of the first-order features (*n* = 18), shape features (*n* = 14), texture features (*n* = 78). Radiomic features were harmonized with the ComBat procedure to compensate for the variability caused by different MR protocols and scanners [34]. Feature normalization was performed using *z*-score.

#### Construction of the radiomic score based radiomic signature

Intra- and inter-observer repeatability for each radiomic feature was evaluated by intraclass correlation coefficient to quantify features stability. The two-way mixed-effects model and the absolute agreement were used to evaluate intra-observer repeatability, and a single rater, absolute agreement, two-way random effect model was used for inter-observer repeatability [35, 36]. Features with intraclass correlation coefficient > 0.75 in both tests were used for the following radiomics analysis. Max-Relevance and Min-Redundancy (mRMR) algorithm was used for feature selection among stable features [37]. The random forest (RF) method was used to construct a radiomic model to predict ER on the training set. Hyperparameter optimization was performed using grid search with tenfold cross-validation to increase model generalizability before building the final model. Radiomic score (Rad-score) for each patient on the training and validation set was calculated using the final radiomic model.

### Assessment of the incremental value of radiomic signature for predicting ER

**Fig. 2:**
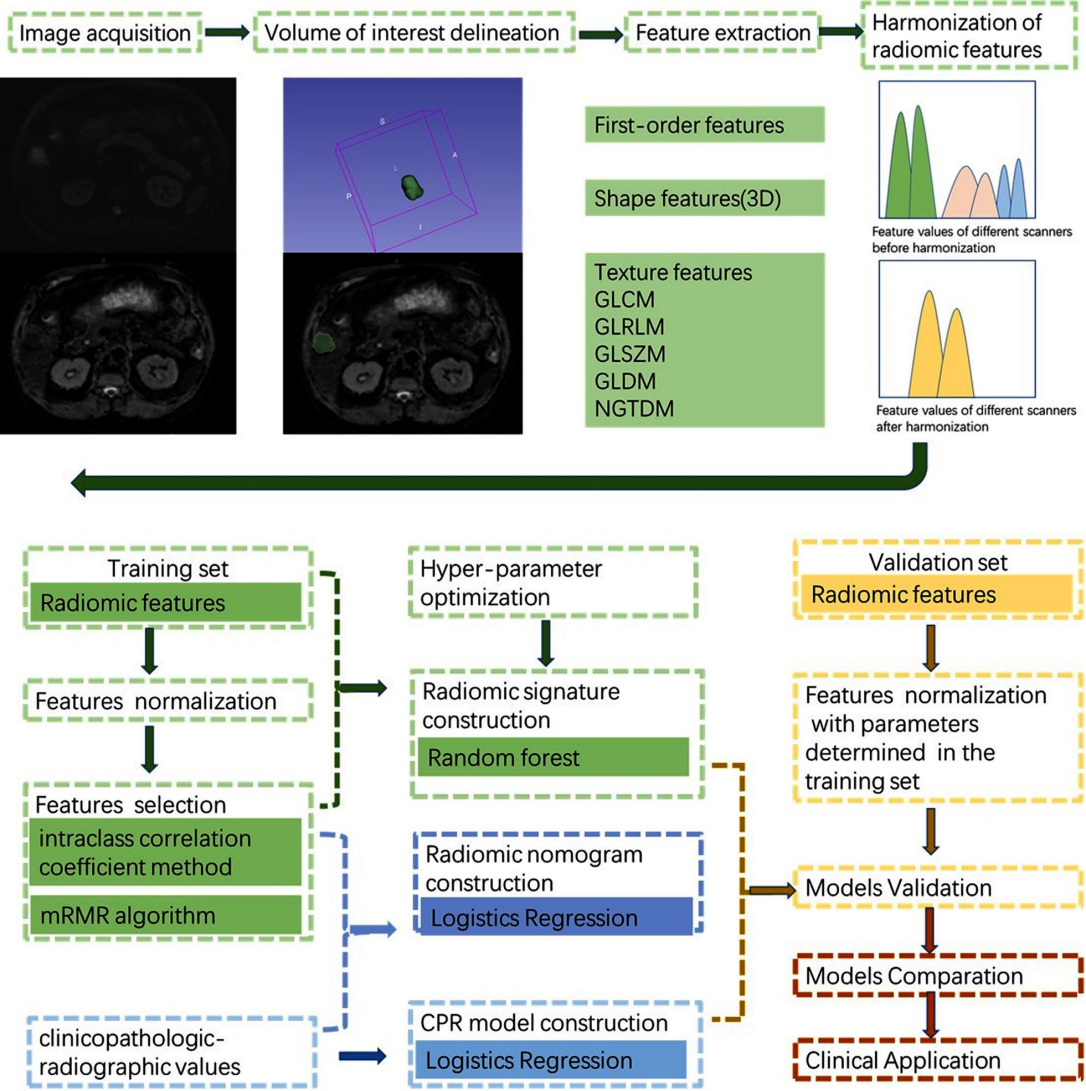
Workflow of this study

A clinicopathological-radiographic (CPR) model based on clinicopathological-radiographic variables and a radiomic nomogram which combined the clinicopathological-radiographic variables and the radiomic signature were constructed through multivariable logistic regression method. Univariate and multivariate backward stepwise LR with the Akaike information criterion (AIC) was used to identify independent clinicopathological-radiographic characteristics. Variables with *p* < 0.1 at univariate analysis were applied to multivariate analysis. The workflow of this study was shown in Fig. 2.

All predictive models were trained on the training set and tested on the independent validation set. Discrimination performances were quantified with the area under the receiver operating characteristic (ROC) curve (AUC) and compared using the DeLong algorithm. Accuracy, sensitivity, specificity of the predictive models was evaluated with the cut-offs determined by maximized Youden index based on the training set. To assess the agreement between the estimated risk and the observed proportion of ER, calibration curves were plotted via bootstrapping with 1000 resamples accompanied by the Hosmer–Lemeshow test. The clinical utility of the predictive models was conducted by calculating the net benefit at different threshold probabilities in the training and validation sets with a decision curve analysis (DCA) [38].

### Evaluation of the association with prognosis

The risk stratification capability of the radiomic signature and nomogram was studied. The model’s cut-off value (the model score at the maximum Youden index) obtained from the training set was used to stratify the low-risk and high-risk groups. Kaplan–Meier survival curves were plotted, and the log-rank test was used to compare the disease-free survival (DFS) and OS. To evaluate whether the radiomic signature could act as an independent predictor for prognosis, univariable and multivariable Cox regression analyses were performed by integrating clinicopathological-radiographic characteristics into models.

### Evaluation of the association with chemotherapy benefit

The survival benefit of chemotherapy within high-risk or low-risk groups defined by different predicting models were analyzed respectively. Kaplan–Meier survival curves and log-rank test were used to compare DFS and OS among patients who either received or did not receive postoperative chemotherapy. In addition, multivariable Cox regression analysis was performed to evaluate the efficacy of adjuvant chemotherapy within different risk groups.

### Clinical outcome and follow-up

All patients regularly underwent computed tomography, MRI, or ultrasonic examination as well as the measurement of serologic tumor markers within 3 months after surgery, thereafter at an interval of 3–6 months. The mean follow-up period was 25.6 months and the median was 23.8 months. ER was defined as local recurrence or distant metastasis that occurred within 1 year after surgery. OS was calculated from the date of surgery to the date of death or the date of the last contact. DFS was calculated from the date of surgery to the first date of local recurrence or distant metastasis, death, or the date of the last contact.

### Statistical analysis

Interobserver agreement for MRI radiographic features was analyzed with the Cohen's Kappa coefficient (Additional file 1: Table S2). To compare variables between different groups, the *X*^2^ test or Fisher exact test was used for categorical variables and the Mann–Whitney U test for continuous variables. Statistical analysis was done with R software (version 3.6.1, www.r-project.org). All *p* values were two-sided. *p* < 0.05 was considered statistically significant.

## Results

### Patient characteristics

In this study, 124 patients including 78 males and 46 females with ICC underwent partial hepatectomy were enrolled. The median OS for the entire cohort was 24.9 months. A total of 47 patients (37.3%) received postoperative adjuvant chemotherapy, with a median DFS of 9.1 months and OS of 32.6 months.

The clinicopathologic and preoperative MR radiographic features in the training (*n* = 87) and validation (*n* = 37) sets were summarized in Table 1. ER rate, DFS, and OS showed no significant difference between the two groups (*p* = 0.847; *p* = 0.56; *p* = 0.44; respectively). But there were significant differences in irregular tumor margin on preoperative MR examinations and major resections (≥ 3 segments according to Couinaud classification) between the training and validation sets (*p* = 0.04, *p* = 0.019; respectively).

**Table 1:** Patient characteristics in the training and validation sets

Characteristics	Training set (*n* = 87)	Validation set (*n* = 37)	*P*
Clinical characteristics			
Sex (Male)	58 (66.7)	20 (54.1)	0.26
Age (years)	56.0 [49.5, 61.5]	56.0 [50.0, 62.0]	0.581
History of HBV infection	68 (78.2)	34 (91.9)	0.115
History of cholelithiasis	17 (19.5)	7 (18.9)	1
Cirrhosis	27 (31.0)	16 (43.2)	0.271
AFP (> 20 ng/ml)	15 (17.2)	5 (13.5)	0.803
CA19-9 (> 1000 U/ml)	16 (18.4)	2 (5.4)	0.11
CEA (> 2.5 ng/ml)	45 (51.7)	21 (56.8)	0.751
MR radiographic characteristics			
Arterial enhancement patterns			0.194
Peripheral rim enhancement	37 (42.5)	11 (29.7)	
Diffuse hyperenhancement	18 (20.7)	13 (35.1)	
Diffuse hypoenhancement	32 (36.8)	13 (35.1)	
Enhancement pattern			0.844
Wash-out pattern	13 (14.9)	6 (16.2)	
Persistent enhancement	12 (13.8)	7 (18.9)	
Gradual enhancement	54 (62.1)	20 (54.1)	
No or minimal enhancement	8 (9.2)	4 (10.8)	
Irregular tumor margin	40 (46.0)	9 (24.3)	**0.04**
Peritumoral enhancement	22 (25.3)	9 (24.3)	1
Peritumoral biliary dilatation	37 (42.5)	13 (35.1)	0.57
Target sign on DWI	49 (56.3)	16 (43.2)	0.255
Multifocal tumor	20 (23.0)	7 (18.9)	0.791
Tumor diameter (cm)	50.0 [36.0, 65.5]	47.0 [33.0, 63.0]	0.533
Pathologic findings			
Surgical margin status (R1)	5 (5.7)	0 (0)	0.322
Macrovascular invasion	28 (32.2)	8 (21.6)	0.332
Microvascular invasion	29 (33.3)	14 (37.8)	0.783
Histologic differentiation			0.808
Well or moderate	39 (44.8)	15 (40.5)	
Poor	48 (55.2)	22 (59.5)	
Lymph node metastasis	37 (42.5)	10 (27.0)	0.154
T stage			0.43
T1a	29 (33.3)	15 (40.5)	
T1b	15 (17.2)	9 (24.3)	
T2	41 (47.1)	13 (35.1)	
T3	2 (2.3)	0 (0.0)	
TNM stage			0.207
IA	26 (29.9)	11 (29.7)	
IB	8 (9.2)	7 (18.9)	
II	15 (17.2)	9 (24.3)	
III	38 (43.7)	10 (27.0)	
Type of surgery			
Extension of hepatectomy			**0.019**
Minor resection	47 (54.0)	29 (78.4)	
Major resection	40 (46.0)	8 (21.6)	
lymphadenectomy	43 (49.4)	15 (40.5)	0.477
Adjuvant therapy			0.331
None	57 (65.5)	20 (54.1)	
Capecitabine	7 (8.0)	7 (18.9)	
Gemcitabine + Capecitabine	13 (14.9)	5 (13.5)	
Gemcitabine + Cisplatin	10 (11.5)	5 (13.5)	
Early recurrence	55 (63.2)	22 (59.5)	0.847

*p* values < 0.05 were considered statistically significant and are shown in bolded font

*HBV* hepatitis B virus, *DWI* diffusion weighted imaging, *AFP* alpha fetoprotein, *CEA* carcinoembryonic antigen, *CA19-9* carbohydrate antigen 19–9, *TNM* tumor, node, metastasis

### Construction and validation of the radiomic signature

A total of 110 radiomic features were extracted from ADC maps for each patient. The top 20 best ranking features associated with ER were selected from 76 stable features to build the radiomic signature (Additional file 1: Table S3). The AUC of the radiomic signature for predicting ER was 0.823(95%CI 0.729–0.917) in the training set and 0.753(95%CI 0.597–0.909) in the validation set.

### Incremental value of radiomic signature

**Fig. 3:**
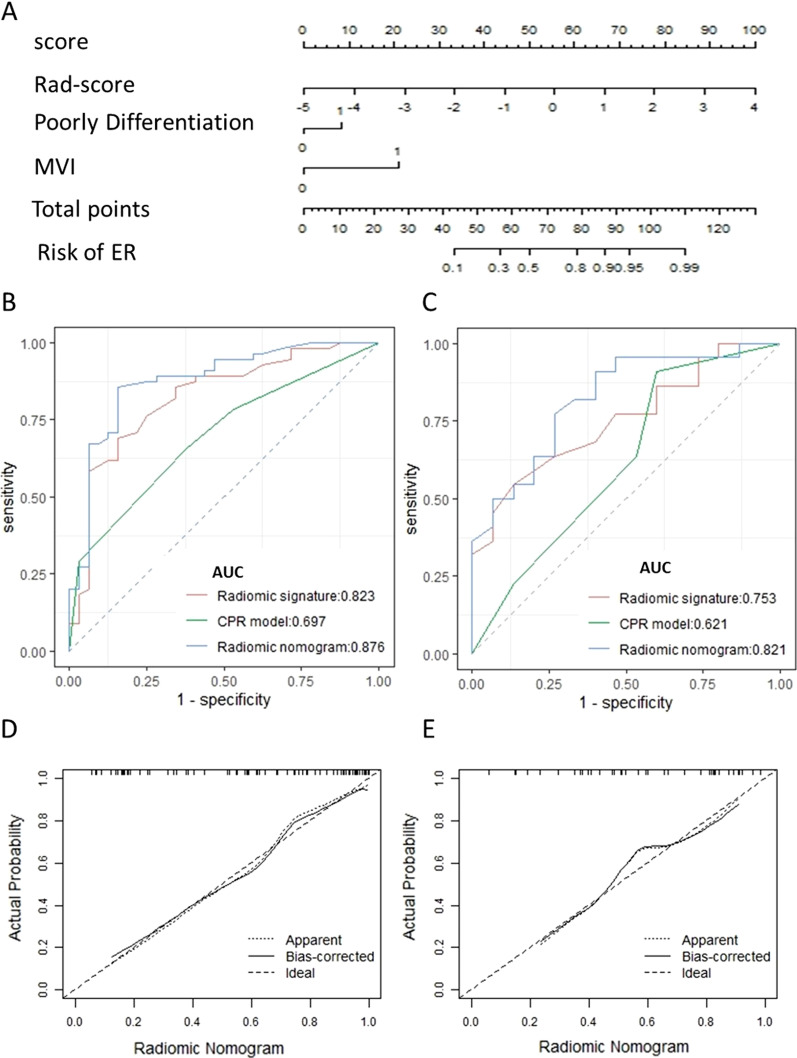
The discrimination performance for predicting early recurrence of different models. Use of the constructed radiomic nomogram to estimate the risk of early recurrence for ICC patients, along with performance assessed by the receiver operating characteristic curves (ROC) and calibration curves. **a** A radiomic nomogram was established based on the training set, with rad-score, poorly differentiation and microvascular invasion (MVI) incorporated. Comparison of ROC curves between clinicopathological and MR radiographic (CPR) model, radiomic signature, and combined radiomic nomogram in the training (**b**) and validation (**c**) sets. Calibration curves of radiomic nomogram in the training (**d**) and validation (**e**) sets

A clinicopathological-radiographic (CPR) model was built with two clinicopathological-radiographic risk factors including poorly differentiation (OR = 3.213, 95%CI 1.283–8.413, *p* = 0.014) and microvascular invasion (OR = 3.180, 95%CI 1.138–10.012, *p* = 0.035), with an AIC score of 109.11(Additional file 1 1 Table S4). A radiomic nomogram was built by incorporating the radiomic signature with the above independent risk factors, and the nomogram yielded a lower AIC score (82.452) (Fig. 3a–c). The calibration curve demonstrated an optimal agreement between the prediction by the nomogram and actual observation both in the training and validation sets (Fig. 3d, e). The Hosmer–Lemeshow test suggested no significant deviation from the ideal fit in both sets (*p* = 0.072; *p* = 0.240; respectively).

The radiomic signature showed comparable discrimination performance for predicting ER compared with CPR model both in the training (AUC, 0.823 vs. 0.697, *p* = 0.06) and validation sets (AUC, 0.753 vs. 0.621, *p* = 0.274) (Table 2). With an AUC of 0.876 (95%CI 0.796–0.955) in the training set and 0.821 (95%CI 0.684–0.959) in the validation set, the combined radiomic nomogram showed significant improvement of discrimination accuracy than CPR model in both sets (*p* = 0.001; *p* = 0.01; respectively, Table 2).

**Table 2:** Predictive performance of radiomic signature, CPR model and radiomic nomogram

Models	AUC (95%CI)	ACC	SEN	SPE	*P*
*Training set*					
CPR model	0.697 (0.592–0.802)	0.644	0.655	0.625	–
Radiomic signature	0.823 (0.729–0.917)	0.747	0.691	0.844	0.06^a^
Radiomic nomogram	0.876 (0.796–0.955)	0.851	0.855	0.844	**0.001**^**b**^
*Validation set*					
CPR model	0.621 (0.434–0.808)	0.649	0.773	0.467	**–**
Radiomic signature	0.753 (0.597–0.909)	0.676	0.591	0.800	0.274^a^
Radiomic nomogram	0.821 (0.684–0.959)	0.757	0.773	0.733	**0.01**^**b**^

*p* values < 0.05 were considered statistically significant and are shown in bolded font

*AUC* the area under the receiver operating characteristic curve, *95%CI* 95% confidence intervals, *ACC* accuracy, *SEN* sensitivity, *SPE* specificity, *CPR model* clinicopathological and MR radiographic model

^a^DeLong test was used to compare the difference of AUC between radiomic signature and CPR model

^b^DeLong test was used to compare the difference of AUC between radiomic nomogram and CPR model

In the training set, the radiomic signature and radiomic nomogram achieved better discrimination accuracy than the TNM stage, with significantly higher AUC values than the TNM stage (TNM, AUC = 0.565, 95%CI 0.448–0.683, *p* < 0.001 for both). A similar result was also found in the validation set (TNM, AUC = 0.505, 95%CI 0.309–0.700; signature vs TNM, *p* = 0.031; nomogram vs TNM, *p* = 0.005; respectively).

**Fig. 4:**
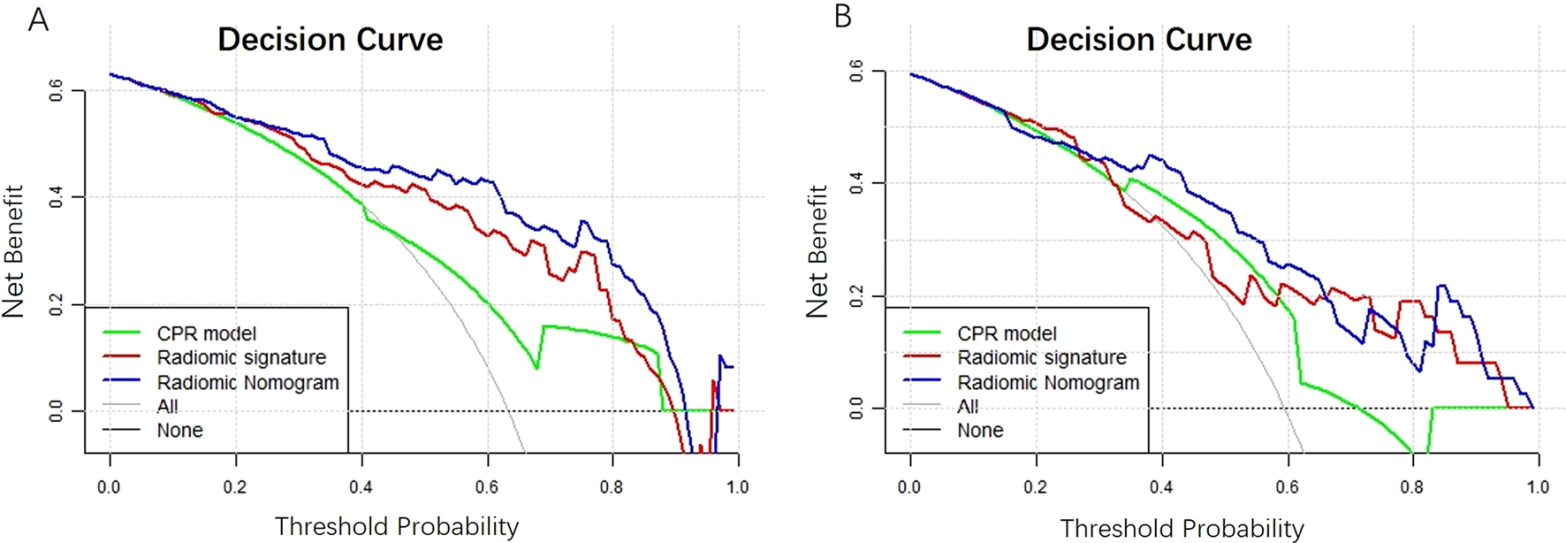
Decision curve analysis. Decision curve analysis for clinicopathological and MR radiographic (CPR) model, radiomic signature, and combined radiomic nomogram in the training (**a**) and validation (**b**) sets

Decision curve analysis showed that the radiomic nomogram gained more net benefits than the “treat all patients” strategy, the “treat none” strategy, as well as radiomic signature and CPR model both in the training and validation sets (Fig. 4).

### Association between radiomic signature and prognosis

**Fig. 5:**
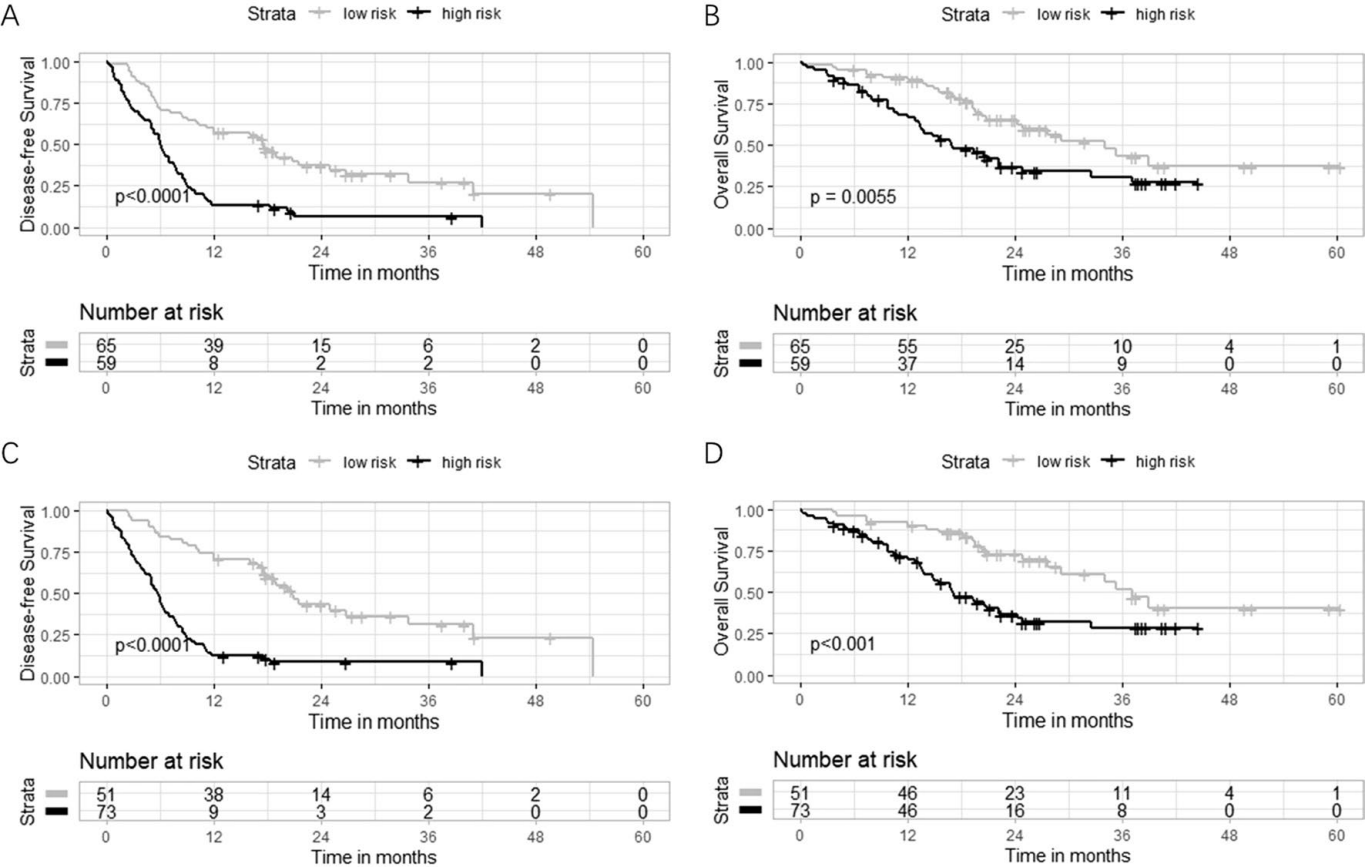
Kaplan–Meier survival analyses for patients with different risk labels. Kaplan–Meier estimates of overall survival (OS) and disease-free survival (DFS) for patients stratified by the radiomic signature (**a**, **b**) and the radiomic nomogram (**c**, **d**) in the entire cohort (*n* = 124)

Based on the training set, we identified the optimum cut-off score of radiomic signature as 0.722. Then, 43 (49.4%) patients in the training set and 16 (43.2%) in the validation set with scores ≥ 0.722 were classified as a high-risk group of ER, and the others as a low-risk group (Additional file 1: Table S5). The two groups defined by the radiomic signature showed significant differences in DFS (*p* < 0.0001) and OS (*p* = 0.0055) (Fig. 5a, b). The radiomic signature remained a powerful and independent prognostic factor for both DFS (HR = 3.112, 95% CI = 1.790–5.410, *p* < 0.0001) and OS (HR = 1.894, 95% CI 1.069–3.356, *p* = 0.029) after adjustment for clinicopathologic-radiographic variables (Table 3 and Additional file 1: Table S6).

**Table 3:** Univariable and multivariable Cox regression analysis of risk factors of overall survival

Characteristics	Univariate analysis			Multivariate analysis		
	HR	95%CI	*p*	HR	95%CI	*p*
Clinical characteristics						
Sex (female)	1.119	0.677–1.849	0.662			
Age (year)	0.993	0.968–1.018	0.577			
History of HBV infection	1.083	0.55–2.131	0.817			
History of cholelithiasis	0.682	0.31–1.501	0.342			
Cirrhosis	1.05	0.617–1.787	0.856			
CA19-9 > 1000 U/mL	1.525	0.793–2.93	0.206			
MR radiographic characteristics						
Arterial enhancement patterns						
Peripheral rim enhancement	ref	ref				
Diffuse hyperenhancement	0.94	0.481–1.839	0.857			
Diffuse hypoenhancement	1.572	0.895–2.762	0.116			
Enhancement pattern						
Wash-out pattern	ref	ref				
Persistent enhancement	1.525	0.542–4.286	0.424			
Gradual enhancement	2.074	0.881–4.882	0.095			
No or minimal enhancement	2.104	0.678–6.527	0.198			
Irregular tumor margin	1.931	1.166–3.198	**0.011**	1.282	0.743–2.212	0.372
Peritumoral enhancement	1.265	0.724–2.209	0.409			
Peritumoral biliary dilatation	2.688	1.625–4.448	** < 0.001**	2.237	1.280–3.911	**0.005**
Target sign on DWI	0.698	0.425–1.146	0.156			
Multifocal tumor	2.052	1.192–3.534	**0.01**	1.132	0.426–3.009	0.803
Tumor diameter(cm)	1.008	0.998–1.018	0.12			
Pathologic findings						
Surgical margin status (R1)	3.157	1.127–8.848	**0.029**	2.871	0.928–8.876	0.067
Macrovascular invasion	2.006	1.193–3.371	**0.009**	1.205	0.449–3.234	0.711
Microvascular invasion	1.594	0.96–2.645	0.071			
Poor differentiation	1.67	0.998–2.795	0.051			
Lymph node metastasis	2.348	1.430–3.857	**0.001**	1.446	0.821–2.550	0.202
T stage						
T1a	Ref					
T1b	1.539	0.720–3.293	0.266	0.914	0.404–2.069	0.83
T2	2.806	1.523–5.171	**0.001**	1.492	0.431–5.163	0.528
T3	2.588	0.338–19.839	0.36	0.758	0.091–6.339	0.798
Adjuvant therapy	0.671	0.391–1.149	0.146			
TACE	0.563	0.176–1.8	0.333			
Ablation therapy	0.489	0.177–1.352	0.168			
Radiomic signature (high risk)	2.009	1.215–3.321	**0.007**	1.894	1.069–3.356	**0.029**

Variables with a *p* value < 0.05 identified on univariable analysis were selected for the multivariable analysis. *p* values < 0.05 were considered statistically significant and are shown in bolded font

*HR* hazard ratio, *95%CI* 95% confidence intervals, *HBV* hepatitis B virus, *DWI* diffusion weighted imaging, *CA19-9* carbohydrate antigen 19–9, *TACE*, transhepatic arterial chemotherapy and embolization

Similarly, the radiomic nomogram stratified 52 (59.8%) patients in the training set and 21(56.8%) in the validation set into a high-risk group with cut-off scores ≥ 0.351, and the others as a low-risk group. Patients with high and low radiomic nomogram scores showed significant differences in DFS (*p* < 0.0001) and OS (*p* < 0.001) (Fig. 5c, d).

### Benefit of adjuvant chemotherapy

**Fig. 6:**
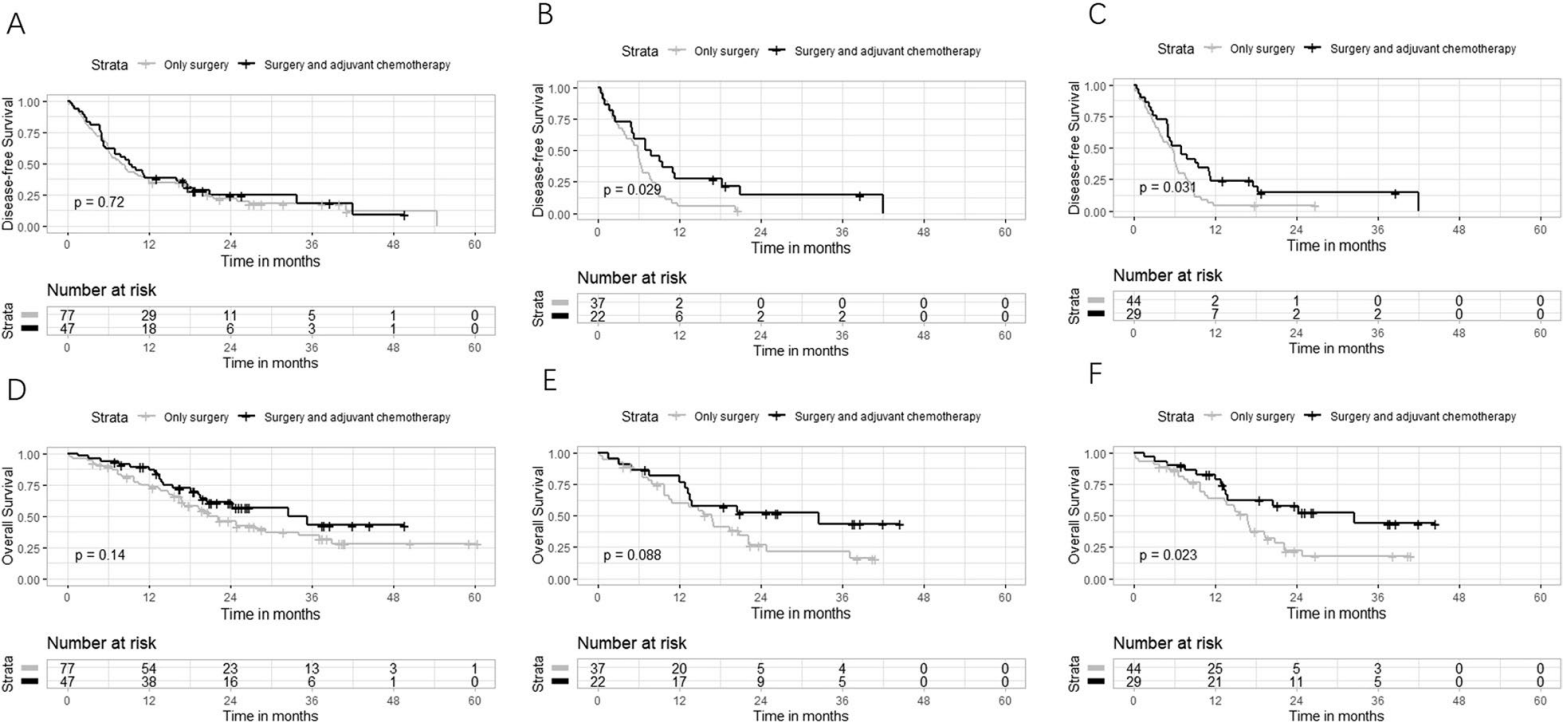
Kaplan–Meier survival analyses for patients with different treatment strategies. Kaplan–Meier curves of DFS for patients in entire cohort (**a**), high-risk group defined by the radiomic signature (**b**), and the high-risk group defined by the radiomic nomogram (**c**). Kaplan–Meier curves of OS for patients in entire cohort (**d**), high-risk group defined by the radiomic signature (**e**), and defined by the radiomic nomogram (**f**)

In the whole cohort, the survival outcomes showed no significant difference between patients who received postoperative adjuvant chemotherapy and patients who did not (Median DFS, 9.1 vs. 8.0 months, *p* = 0.72; Median OS, 32.6 vs. 20.9 months, *p* = 0.14) (Additional file 1: Table S7, Fig. 6a, d). Among patients who received adjuvant chemotherapy, patients treated with capecitabine, gemcitabine combined with capecitabine, or gemcitabine combined with cisplatin show no significant difference of OS (*p* = 0.29) and DFS (*p* = 0.18).

Adjuvant chemotherapy improved prognosis in high-risk patients defined by the radiomic signature (DFS, *p* = 0.029, Fig. 6b; OS, *p* = 0.088, Fig. 6e) and defined by the nomogram (DFS, *p* = 0.031, Fig. 6c; OS, *p* = 0.023, Fig. 6f), whereas poor chemotherapy efficacy was detected in low-risk patients (Additional file 1: Tables S8–S15, Table 4). The CPR model failed for predicting the benefit of adjuvant chemotherapy (Table 4).

**Table 4:** The survival benefits of postoperative adjuvant chemotherapy for patients in different risk groups

Models	Endpoints (months)	High-risk group			Low-risk group		
		Only surgery	Surgery + adjuvant chemotherapy	*p*	Only surgery	Surgery + adjuvant chemotherapy	*p*
Radiomic signature	DFS	5.9	7.5	**0.029**	19.0	11.2	0.32
	OS	16.8	32.6	0.088	34.2	35.3	0.82
CPR model	DFS	6.4	6.4	0.64	20.9	19.2	0.43
	OS	19.3	24.3	0.61	29.3	NA	0.091
Radiomic nomogram	DFS	5.6	7.0	**0.031**	20.9	19.2	0.43
	OS	16.8	32.6	**0.023**	39.0	35.2	0.62

Median DFS and OS were calculated using Kaplan Meier method and the benefits of different treatment strategies were compared by two-sided log-rank tests. *p* values < 0.05 were considered statistically significant and are shown in bolded font

*CPR model* clinicopathological and MR radiographic model, *DFS* disease-free survival, *OS* overall survival

## Discussion

In this study, we developed and validated a preoperative DWI-based radiomic signature for individualized prediction of ER in patients with ICC who underwent partial hepatectomy. This radiomic signature succeeded to stratify patients into distinct survival outcomes according to their risk index of ER, and could serve as an effective tool for screening patients who might benefit from postoperative adjuvant chemotherapy. By combining clinicopathologic-radiographic predictors and radiomic signature, the integrated radiomic nomogram had a much-improved performance for predicting ER and adjuvant chemotherapy benefits compared with the CPR model. These results demonstrated that the radiomic signature provided useful and complementary information about the prognosis of tumors beyond currently known clinicopathologic-radiographic predictors.

ICC is characterized by varying degrees of stromal desmoplasia [39]. A previous study proved that ICCs with tumors that exhibited an abundant fibrous stroma had increased rates of perineural invasion, lymphatic invasion, and worsened prognosis than those characterized by scanty fibrous stroma [40]. Lee et al. found that the area within the tumor without diffusion restriction on DWI is correlated with the areas of scattered neoplastic cells in dense fibrous stroma, and volume ratios of diffusion restriction within the tumor is an independent prognostic factor for OS of ICC [23]. Hence DWI is considered as an effective prognostic tool for ICC by characterizing the spatial distribution of tumor cellularity and fibrous stroma. However, simply calculating ADC values of the entire tumor or volume ratios of diffusion restriction is difficult to provide detailed information about the characteristics of fibrous stroma within the tumor, since the tumor is heterogeneous and fibrous stroma is not uniformly mixed throughout the whole volume [39]. In fact, intratumoral heterogeneity is often not macroscopic on imaging [41]. Based on quantitative analyses of high-dimension image features, the radiomics signature could provide more powerful and accurate interpreting of intratumoral heterogeneity and it has become a useful imaging marker to predict the prognosis of malignant tumors [18].

Based on the results of a multicenter III trial conducted in the UK, the American Society of Clinical Oncology (ASCO) recommends postoperative adjuvant chemotherapy as the standard of care [11, 42]. However, the survival benefits of chemotherapy vary among patients. The biological heterogeneity of ICC perhaps explains the different efficacy of adjuvant chemotherapy in unselected patients. A meta-analysis involving 6,712 patients suggested the potential benefit for adjuvant therapy in patients with margin-positive disease (OR = 0.36, *p* = 0.002) [43]. On the other hand, according to the subset analysis limited to node-positive patients in a randomized clinical trial, the chemotherapy group had a worse OS than the observation group (median OS, 28.3 vs. 28.8 month) [15].

Predicting biomarkers or models to personalize risk–benefit evaluation of chemotherapy will improve patient outcomes and avoid unnecessary toxicities and economic burdens for patients with non-chemotherapy benefits. In this study, we established DWI-based radiomic signature as a strong indicator for postoperative adjuvant chemotherapy, i.e., high-risk patients could benefit from chemotherapy while low-risk could not. By combining clinicopathologic-radiologic features and radiomic signature, we showed that an integrated radiomic nomogram had a better ability to identify patients who may benefit from chemotherapy than did either alone. Though the process of radiomics tends to be particularly tedious and time-consuming so far, the development of fast segmentation tool and easy-to-use software interface will facilitate the application of radiomic signature in prognostic assessment and clinical decisions in the future [44].

There were several limitations in this study. First, this is a single-center retrospective study, and the validation set is of small size, external validation is needed to assess the generalizability of the reported findings. Second, a large number of patients who did not complete at least 1 year of follow-up were excluded from this study. The reasons for the incomplete follow-up may include that some patients chose to undergo postoperative surveillance and treatment in other medical institutions, and/or their contact information had changed. Although the exclusion of these patients may cause potential bias, it may have no big influence on the outcomes of our study since the prognosis of patients included in our final cohort was comparable with that of other similar cohorts which were from different regions of the same country (ER rate, 62.1% vs. 63.6% [19]; OS, 24.9 vs. 21.0 months [6]). Third, MR imaging data in our study were acquired on different scanners with similar protocols, which may affect radiomic feature values. To compensate for the technical variability of radiomic features, we performed MRI signal intensity normalization and voxel size resampling prior to radiomic feature extraction, and realigned radiomic feature distributions and removes the scanner effect by using ComBat harmonization before designing the model [34]. Hence, our radiomic model may have a good generalization when applied to data from different centers related to different MR scanners. Fourth, the use of adjuvant chemotherapy was not randomly assigned to patients as a result of its retrospective nature. We used multivariable Cox regression analysis to evaluate the efficacy of adjuvant chemotherapy after adjusted for common clinicopathologic factors and treatments.

In conclusion, the DWI-based radiomic signature could predict early recurrence risk for patients with ICC after partial hepatectomy, stratify survival outcomes, and identify patients who are most likely to benefit from postoperative adjuvant chemotherapy. The DWI-based radiomic signature could help to guide individualized therapeutic selection for ICC patients.

## Supplementary Information

**Additional file 1**. ** Table S1**. MRI sequence parameters.**Table S2**. Inter-observer agreement for MRI radiographic features. **Table S3**. Inter- and intra-observer repeatability for radiomic features.** Table S4**. Logistics regression analysis of risk factors of early recurrence.** Table S5**. Patient Characteristics in the high- and low-risk groups defined by the radiomic signature.** Table S6**. Cox regression analysis of risk factors of DFS.** Table S7**. Patient Characteristics according to the postoperative adjuvant chemotherapy.** Table S8**. Cox regression analysis of DFS within the high-risk group defined by the radiomic signature.** Table S9**. Cox regression analysis of OS within the high-risk group defined by the radiomic signature.** Table S10**. Cox regression analysis of DFS within the low-risk group defined by the radiomic signature.** Table S11**. Cox regression analysis of OS within the low-risk group defined by the radiomic signature.** Table S12**. Cox regression analysis of DFS within the high-risk group defined by the radiomic nomogram.** Table S13**. Cox regression analysis of OS within the high-risk group defined by the radiomic nomogram.** Table S14**. Cox regression analysis of DFS within the low-risk group defined by the radiomic nomogram.** Table S15**. Cox regression analysis of OS within the low-risk group defined by the radiomic nomogram.

## Data Availability

The datasets used and/or analyzed during the current study are available from the corresponding author on reasonable request.

## Abbreviations

ADC: Apparent diffusion coefficient
AFP: Alpha fetoprotein
AIC: Akaike information criterion
AJCC: American joint committee on cancer
ASCO: The American society of clinical oncology
AUC: The area under the receiver operating characteristic curve
CA19-9: Carbohydrate antigen 19–9
CEA: Carcinoembryonic antigen
CPR: Clinicopathologic-radiographic
DCA: Decision curve analysis
DFS: Disease-free survival
DWI: Diffusion-weighted images
ER: Early recurrence
IBIS: The imaging biomarker standardization initiative
ICC: Intrahepatic cholangiocarcinoma
MR: Magnetic resonance
MRI: Magnetic resonance imaging
mRMR: Max-relevance and min-redundancy
OS: Overall survival
RF: Random forest
ROC: Receiver operating characteristic
ROI: Region of interest
TNM: Tumor, node, and metastasis
