# Supplementary material for: DWI-based radiomic signature: potential role for individualized adjuvant chemotherapy in intrahepatic cholangiocarcinoma after partial hepatectomy
Source: Insights Imaging. 2022 Mar 4;13:37. doi: 10.1186/s13244-022-01179-7 (PMC8897536; doi:10.1186/s13244-022-01179-7)
Supplement: Supplementary file 1 — Additional file 1. Table S1. MRI sequence parameters.Table S2. Inter-observer agreement for MRI radiographic features. Table S3. Inter- and intra-observer repeatability for radiomic features. Table S4. Logistics regression analysis of risk factors of early recurrence. Table S5. Patient Characteristics in the high- and low-risk groups defined by the radiomic signature. Table S6. Cox regression analysis of risk factors of DFS. Table S7. Patient Characteristics according to the postoperative adjuvant chemotherapy. Table S8. Cox regression analysis of DFS within the high-risk group defined by the radiomic signature. Table S9. Cox regression analysis of OS within the high-risk group defined by the radiomic signature. Table S10. Cox regression analysis of DFS within the low-risk group defined by the radiomic signature. Table S11. Cox regression analysis of OS within the low-risk group defined by the radiomic signature. Table S12. Cox regression analysis of DFS within the high-risk group defined by the radiomic nomogram. Table S13. Cox regression analysis of OS within the high-risk group defined by the radiomic nomogram. Table S14. Cox regression analysis of DFS within the low-risk group defined by the radiomic nomogram. Table S15. Cox regression analysis of OS within the low-risk group defined by the radiomic nomogram. [file 13244_2022_1179_MOESM1_ESM.docx]

**ADDITIONAL FILE 1**

**Diffusion-weighted Imaging-based Radiomic Signature: Predicting Adjuvant Chemotherapy Benefit in Intrahepatic Cholangiocarcinoma**

**Content:**

[Additional file 1 Tables 2](#_Toc489355122)

[Additional file 1: Table S1 MRI sequence parameters 2](#_Toc489355123)

[Additional file 1: Table S2 Inter-observer agreement for MRI radiographic features. 3](#_Toc489355124)

[Additional file 1: Table S3 Inter- and intra-observer repeatability for radiomic features 4](#_Toc489355125)

[Additional file 1: Table S4 Logistics regression analysis of risk factors of early recurrence 5](#_Toc489355133)

*Additional file 1: Table S5* [*Patient Characteristics in the high- and low-risk groups defined by the radiomic signature. 6*](#_Toc489355134)

[*Additional file 1: Table S6 Cox regression analysis of risk factors of DFS. 8*](#_Toc489355135)

[*Additional file 1: Table S7 Patient Characteristics according to the postoperative adjuvant chemotherapy. 9*](#_Toc489355136)

[*Additional file 1: Table S8 Cox regression analysis of DFS within the high-risk group defined by the radiomic signature. 11*](#_Toc489355137)

[*Additional file 1: Table S9 Cox regression analysis of OS within the high-risk group defined by the radiomic signature 12*](#_Toc489355138)

[*Additional file 1: Table S10 Cox regression analysis of DFS within the low-risk group defined by the radiomic signature. 13*](#_Toc489355137)

[*Additional file 1: Table S11 Cox regression analysis of OS within the low-risk group defined by the radiomic signature 14*](#_Toc489355138)

[*Additional file 1: Table S12 Cox regression analysis of DFS within the high-risk group defined by the radiomic nomogram. 15*](#_Toc489355137)

[*Additional file 1: Table S13 Cox regression analysis of OS within the high-risk group defined by the radiomic nomogram 16*](#_Toc489355138)

[*Additional file 1: Table S14 Cox regression analysis of DFS within the low-risk group defined by the radiomic nomogram. 17*](#_Toc489355137)

[*Additional file 1: Table S15 Cox regression analysis of OS within the low-risk group defined by the radiomic nomogram 18*](#_Toc489355138)

| Additional file 1: Table S1 MR Imaging Parameters | | | | | | | |
| --- | --- | --- | --- | --- | --- | --- | --- |
| Sequence | Field Strength | Matrix | Section Thickness(mm) | Intersection Gap(mm) | Repetition Time(msec) | Echo Time(msec) | Flip Angle (degrees) |
| Coronal FIESTA; Coronal T2-weighted SSFSE /HASTE  breath-hold | 1.5T(Optima MR360,GE) | 224×160 | 6 | 1 | 3.44 | 1.48 | 55 |
|  | 3.0T(Discovery MR750,GE) | 224×384 | 6 | 1 | 1800 | 70 | 90 |
|  | 3.0T(MAGNETOM Skyra, Siemens) | 256×320 | 5 | 0-1 | 1100 | 87 | 155 |
| T1-weighted LAVA/ VIBE  breath-hold | 1.5T(Optima MR360,GE) | 256×160 | 5 | 0 | 6.28 | 3.16 | 12 |
|  | 3.0T(Discovery MR750,GE) | 288×192 | 5 | 0 | 3.7 | 1.1 | 12 |
|  | 3.0T(MAGNETOM Skyra, Siemens) | 352×200 | 3 | 0 | 5.77 | 2.46 | 12 |
| axial T2-weighted FSE/TSE respiratory-triggered | 1.5T(Optima MR360,GE) | 320×192 | 6 | 1 | 5454-7500 | 52.5-89.4 | 90 |
|  | 3.0T(Discovery MR750,GE) | 320×320 | 6 | 1 | 8571-9000 | 60-85 | 90 |
|  | 3.0T(MAGNETOM Skyra, Siemens) | 320×320 | 6 | 1 | 2957-5023 | 79-89 | 106-130 |
| diffusion weighted images  single-shot echo-planar sequence | 1.5T(Optima MR360,GE) | 130×160 | 6-8 | 1 | 5050-9000 | 52.8-71.4 | 90 |
|  | 3.0T(Discovery MR750,GE) | 128×128 | 5-6 | 1 | 3157-7500 | 52.9-82 | 90 |
|  | 3.0T(MAGNETOM Skyra, Siemens) | 128×104 | 6 | 0-1.2 | 4800-6500 | 50-62 | 90 |
| T1-weighted LAVA/VIBE(Dynamic enhanced liver MRI)^1^ | 1.5T(Optima MR360,GE) | 256×160 | 5 | 0 | 6.05 | 3.12 | 12 |
|  | 3.0T(Discovery MR750,GE) | 288×160 | 5 | 0 | 3.7 | 1.7 | 12 |
|  | 3.0T(MAGNETOM Skyra, Siemens) | 352×200 | 3.5 | 0 | 3.19 | 1.2 | 9 |
| 1 For dynamic imaging, 0.1 mmol/kg of Multihance (Bracco Imaging, Milan, Italy) were injected via a high-pressure injector (2.5 mL/s), followed by 30mL saline immediately. Dynamic study in the hepatic arterial phase, portal venous, and delayed phases were acquired at 20, 70, and 240 seconds after the injection respectively.  Abbreviations：FIESTA, fast imaging employing steady-state acquisition; SSFSE, single shot fast spin echo; HASTE, half-Fourier acquisition single shot turbo spin echo; LAVA, liver acquisition with volume acceleration; VIBE, volumetric interpolated breath-hold examination; FSE, fast spin echo; TSE, turbo spin echo. | | | | | | | |

| Additional file 1: Table S2 Inter-observer agreement for MRI radiographic features. | |
| --- | --- |
| Radiographic features | Intra-observer agreement |
| Arterial enhancement patternsa | 0.878 |
| Enhancement pattern a | 0.856 |
| Irregular tumor margin a | 0.884 |
| Peritumoral enhancement a | 0.938 |
| Peritumoral biliary dilatationa | 0.950 |
| Target sign on DWI a | 0.919 |
| Multifocal tumora | 0.977 |
| Tumor diameterb | 0.977 |
| a, Cohen's Kappa coefficient was used to evaluate the interobserver agreement; b, intraclass correlation coefficient was used to evaluate the interobserver agreement. | |

| Additional file 1: Table S3 Inter- and intra-observer repeatability for radiomic features included in the radiomics model. | | | |
| --- | --- | --- | --- |
| Radiomics features | Inter-observer agreement ^a^ | Intra-observer agreement ^a^ | |
| firstorder_10Percentile | 0.975 | | 0.964 |
| firstorder_Minimum | 0.934 | | 0.943 |
| glcm_ClusterProminence | 0.979 | | 0.911 |
| glcm_ClusterShade | 0.952 | | 0.952 |
| glcm_Correlation | 0.942 | | 0.952 |
| glcm_Idmn | 0.918 | | 0.884 |
| glcm_Idn | 0.962 | | 0.957 |
| glcm_Imc1 | 0.912 | | 0.922 |
| glcm_InverseVariance | 0.995 | | 0.996 |
| gldm_DependenceNonUniformity | 0.997 | | 0.997 |
| gldm_DependenceNonUniformityNormalized | 0.984 | | 0.985 |
| gldm_DependenceVariance | 0.962 | | 0.959 |
| gldm_SmallDependenceHighGrayLevelEmphasis | 0.903 | | 0.931 |
| gldm_SmallDependenceLowGrayLevelEmphasis | 0.78 | | 0.779 |
| glrlm_GrayLevelNonUniformity | 0.937 | | 0.935 |
| glrlm_LongRunHighGrayLevelEmphasis | 0.781 | | 0.78 |
| glrlm_RunLengthNonUniformity | 0.903 | | 0.91 |
| glrlm_ShortRunLowGrayLevelEmphasis | 0.773 | | 0.76 |
| ngtdm_Busyness | 0.823 | | 0.833 |
| ngtdm_Contrast | 0.947 | | 0.941 |
| a, intraclass correlation coefficient was used to evaluate the inter- and intra-observer repeatability for each radiomic feature. | | | |

| Additional file 1: Table S4 Univariable and multivariable logistics regression analysis of risk factors of early recurrence. | | | | | | |  |
| --- | --- | --- | --- | --- | --- | --- | --- |
| Characteristics | Univariate Analysis | | | Multivariate Analysis | | |  |
|  | OR | 95%CI | P | HR | 95%CI | P |  |
| Clinical Characteristics |  |  |  |  |  |  |  |
| Sex | 2.381 | 0.909-6.824 | 0.088 |  |  |  |  |
| age (year) | 0.988 | 0.943-1.034 | 0.605 |  |  |  |  |
| History of HBV infection | 1.333 | 0.46-3.752 | 0.587 |  |  |  |  |
| History of cholelithiasis | 1.37 | 0.404-5.436 | 0.627 |  |  |  |  |
| cirrhosis | 1.243 | 0.486-3.33 | 0.655 |  |  |  |  |
| CA19-9＞1000U/mL | 0.963 | 0.319-3.112 | 0.947 |  |  |  |  |
| MR Radiographic Characteristics | |  |  |  |  |  |  |
| Arterial enhancement patterns |  |  |  |  |  |  |  |
| Peripheral rim enhancement | ref | ref |  |  |  |  |  |
| Diffuse hyperenhancement | 0.609 | 0.192-1.908 | 0.393 |  |  |  |  |
| Diffuse hypoenhancement | 1.556 | 0.568-4.415 | 0.395 |  |  |  |  |
| Enhancement pattern |  |  |  |  |  |  |  |
| Wash-out pattern | ref | ref |  |  |  |  |  |
| Persistent enhancement | 1.163 | 0.647-18.067 | 0.164 |  |  |  |  |
| Gradual enhancement | 1.001 | 0.798-10.098 | 0.116 |  |  |  |  |
| No or minimal enhancement | 18.036 | 0.000-NA | 0.990 |  |  |  |  |
| Irregular tumor margin | 2.129 | 0.877-5.376 | 0.1 |  |  |  |  |
| Peritumoral enhancement | 1.339 | 0.491-3.925 | 0.577 |  |  |  |  |
| Peritumoral biliary dilatation | 2.121 | 0.864-5.46 | 0.107 |  |  |  |  |
| Target sign on DWI | 1.005 | 0.414-2.419 | 0.992 |  |  |  |  |
| Multifocal tumor | 1.48 | 0.522-4.621 | 0.475 |  |  |  |  |
| Tumor diameter(cm) | 1.002 | 0.983-1.023 | 0.811 |  |  |  |  |
| Pathologic findings |  |  |  |  |  |  |  |
| Surgical margin status (R1) | 0.865 | 0.136-6.848 | 0.878 |  |  |  |  |
| Macrovascular invasion | 2.206 | 0.839-6.333 | 0.121 |  |  |  |  |
| Microvascular invasion | 3.115 | 1.156-9.459 | 0.032 | 3.180 | 1.138-10.012 | **0.035** |  |
| Poor differentiation | 3.158 | 1.295-8.007 | 0.013 | 3.213 | 1.283-8.413 | **0.014** |  |
| Lymph node metastasis | 2.121 | 0.864-5.46 | 0.107 |  |  |  |  |
| T stage |  |  |  |  |  |  |  |
| T1a | Ref |  |  |  |  |  |  |
| T1b | 0.698 | 0.195-2.500 | 0.577 |  |  |  |  |
| T2 | 1.316 | 0.481-3.588 | 0.589 |  |  |  |  |
| T3 | 0.611 | 0.023-16.515 | 0.737 |  |  |  |  |
| Adjuvant therapy | 1.008 | 0.405-2.567 | 0.987 |  |  |  |  |
| Variables with a p value <0.1 identified on univariable analysis were selected for the multivariable analysis.  Abbreviations: HR, hazard ratio; 95%CI, 95% confidence intervals. HBV, hepatitis B virus; DWI, diffusion weighted imaging; CA19-9, carbohydrate antigen 19-9，TACE，transhepatic arterial chemotherapy and embolization. | | | | | | |  |
|  | | | | | | |  |

| Additional file 1: Table S5 Patient Characteristics in the high- and low-risk groups defined by the radiomic signature. | | | |
| --- | --- | --- | --- |
| Characteristics | Low-risk (n=65) | High-risk(n=59) | *P* |
| Clinical Characteristics |  |  |  |
| Sex(Male) | 46(70.8) | 32(54.2) | 0.086 |
| Age(years) | 56.0[50.0, 62.0] | 56.0[50.0, 61.5] | 0.984 |
| History of HBV infection | 54 (83.1) | 48 (81.4) | 0.988 |
| history of cholelithiasis | 9 (13.8) | 10 (16.9) | 0.819 |
| Cirrhosis | 25 (38.5) | 18 (30.5) | 0.459 |
| AFP (>20ng/ml) | 10 (15.4) | 10(16.9) | 1 |
| CA19-9 (>1000U/ml) | 10 (15.4) | 8 (13.6) | 0.974 |
| CEA (>2.5ng/ml) | 32 (49.2) | 34 (57.6) | 0.45 |
| MR Radiographic Characteristics |  |  |  |
| Arterial enhancement patterns |  |  | 0.732 |
| Peripheral rim enhancement | 25 (38.5) | 23 (39.0) |  |
| Diffuse hyperenhancement | 18 (27.7) | 13 (22.0) |  |
| Diffuse hypoenhancement | 22 (33.8) | 23 (39.0) |  |
| Enhancement pattern |  |  | **0.007** |
| Wash-out pattern | 13 (20.0) | 6 (10.2) |  |
| Persistent enhancement | 15 (23.1) | 4 (6.8) |  |
| Gradual enhancement | 34 (52.3) | 40 (67.8) |  |
| No or minimal enhancement | 3 (4.6) | 9 (15.3) |  |
| Irregular tumor margin | 21 (32.3) | 28 (47.5) | 0.124 |
| Peritumoral enhancement | 12 (18.5) | 19 (32.2) | 0.119 |
| Peritumoral biliary dilatation | 25 (38.5) | 25 (42.4) | 0.795 |
| Target sign on DWI | 30 (46.2) | 35 (59.3) | 0.198 |
| Multifocal tumor | 10 (15.4) | 17 (28.8) | 0.111 |
| Tumor diameter(cm) | 40.0[30.0, 52.0] | 61.0[47.5, 79.0] | **<0.001** |
| Pathologic findings |  |  |  |
| Surgical margin status(R1) | 2 (3.1) | 3 (5.1) | 0.912 |
| Macrovascular invasion | 17 (26.2) | 19(32.2) | 0.587 |
| Microvascular invasion | 21 (32.3) | 22 (37.3) | 0.694 |
| Histologic differentiation |  |  | 0.025 |
| Well or moderate | 35 (53.8) | 19 (32.2) |  |
| Poor | 30 (46.2) | 40 (67.8) |  |
| Lymph node metastasis | 21 (32.3) | 26 (44.1) | 0.245 |
| T stage |  |  | **0.017** |
| T1a | 30 (46.2) | 14 (23.7) |  |
| T1b | 8 (12.3) | 16 (27.1) |  |
| T2 | 27 (41.5) | 27 (45.8) |  |
| T3 | 0 (0.0) | 2 (3.4) |  |
| TNM stage |  |  | 0.067 |
| ⅠA | 26 (40.0) | 11 (18.6) |  |
| ⅠB | 6 (9.2) | 9 (15.3) |  |
| Ⅱ | 12 (18.5) | 12 (20.3) |  |
| Ⅲ | 21 (32.3) | 27 (45.8) |  |
| Type of surgery |  |  |  |
| Extension of hepatectomy |  |  | 0.326 |
| Minor resection | 43 (66.2) | 33 (55.9) |  |
| Major resection | 22 (33.8) | 26 (44.1) |  |
| lymphadenectomy | 31 (47.7) | 27 (45.8) | 0.972 |
| Adjuvant therapy | 25 (38.5) | 22 (37.3) | 1 |
| Early recurrence | 26 (40.0) | 51 (86.4) | **<0.001** |
| Rad-score | -0.10 [-0.86, 0.41] | 1.54 [1.16, 2.56] | **<0.001** |
| Abbreviations：HBV, hepatitis B virus; DWI, diffusion weighted imaging; AFP, alpha fetoprotein; CEA, carcinoembryonic antigen; CA19-9, carbohydrate antigen 19-9. | | | |

| Additional file 1: Table S6 Univariable and multivariable Cox regression analysis of risk factors of disease-free survival. | | | | | | |
| --- | --- | --- | --- | --- | --- | --- |
| Characteristics | Univariate Analysis | | | Multivariate Analysis | | |
|  | HR | 95%CI | P | HR | 95%CI | P |
| Clinical Characteristics |  |  |  |  |  |  |
| Sex(female) | 1.24 | 0.825-1.862 | 0.301 |  |  |  |
| Age (year) | 0.984 | 0.963-1.006 | 0.153 |  |  |  |
| History of HBV infection | 1.093 | 0.638-1.871 | 0.747 |  |  |  |
| History of cholelithiasis | 0.682 | 0.31-1.501 | 0.342 |  |  |  |
| Cirrhosis | 0.984 | 0.644-1.505 | 0.942 |  |  |  |
| CA19-9＞1000U/mL | 1.292 | 0.743-2.245 | 0.364 |  |  |  |
| MR Radiographic Characteristics | | |  |  |  |  |
| Arterial enhancement patterns |  |  |  |  |  |  |
| Peripheral rim enhancement | ref | ref |  |  |  |  |
| Diffuse hyperenhancement | 1.026 | 0.606-1.738 | 0.925 |  |  |  |
| Diffuse hypoenhancement | 1.326 | 0.838-2.1 | 0.228 |  |  |  |
| Enhancement pattern |  |  |  |  |  |  |
| Wash-out pattern | ref | ref |  |  |  |  |
| Persistent enhancement | 1.426 | 0.654-3.109 | 0.372 | 0.622 | 0.247-1.570 | 0.315 |
| Gradual enhancement | 1.741 | 0.911-3.327 | 0.093 | 1.867 | 1.136-3.071 | **0.014** |
| No or minimal enhancement | 3.332 | 1.458-7.615 | 0.004 | 2.086 | 0.891-4.882 | 0.09 |
| Irregular tumor margin | 1.575 | 1.047-2.37 | 0.029 | 1.677 | 0.817-3.441 | 0.159 |
| Peritumoral enhancement | 1.344 | 0.855-2.111 | 0.2 |  |  |  |
| Peritumoral biliary dilatation | 1.638 | 1.088-2.467 | 0.018 | 2.790 | 1.159-6.719 | **0.022** |
| Target sign on DWI | 0.95 | 0.637-1.417 | 0.801 |  |  |  |
| Multifocal tumor | 1.926 | 1.211-3.061 | 0.006 | 1.102 | 0.686-1.771 | 0.688 |
| Tumor diameter(cm) | 1.01 | 1.002-1.018 | 0.016 | 1.571 | 0.969-2.547 | 0.067 |
| Pathologic findings |  |  |  |  |  |  |
| Surgical margin status (R1) | 1.524 | 0.558-4.159 | 0.411 |  |  |  |
| Macrovascular invasion | 1.561 | 1.007-2.42 | 0.046 | 1.048 | 0.443-2.482 | 0.915 |
| Microvascular invasion | 1.83 | 1.211-2.765 | 0.004 | 1.001 | 0.989-1.013 | 0.842 |
| Poor differentiation | 1.552 | 1.031-2.336 | 0.035 | 0.573 | 0.284-1.156 | 0.12 |
| Lymph node metastasis | 1.412 | 0.94-2.122 | 0.097 |  |  |  |
| T stage |  |  |  |  |  |  |
| T1a | Ref |  |  |  |  |  |
| T1b | 0.992 | 0.55-1.789 | 0.979 | 1.797 | 0.602-5.364 | 0.294 |
| T2 | 1.769 | 1.122-2.789 | 0.014 | 0.513 | 0.099-2.654 | 0.426 |
| T3 | 1.378 | 0.329-5.764 | 0.661 | 1.248 | 0.793-1.963 | 0.338 |
| Adjuvant therapy | 0.927 | 0.613-1.401 | 0.719 |  |  |  |
| Radiomics signature (high risk) | 2.741 | 1.811-4.149 | <0.0001 | 3.112 | 1.790-5.410 | **<0.0001** |
| Variables with a p value <0.05 identified on univariable analysis were selected for the multivariable analysis.  Abbreviations: HR, hazard ratio; 95%CI, 95% confidence intervals. HBV, hepatitis B virus; DWI, diffusion weighted imaging; CA19-9, carbohydrate antigen 19-9，TACE，transhepatic arterial chemotherapy and embolization. | | | | | | |

| Additional file 1: Table S7 Patient Characteristics according to the postoperative adjuvant chemotherapy. | | | |
| --- | --- | --- | --- |
| Characteristics | Only surgery(n=77) | Adjuvant chemotherapy (n=47) | *P* |
| Clinical Characteristics |  |  |  |
| Sex(Male) | 52 (67.5) | 26 (55.3) | 0.24 |
| Age(years) | 56.00 [51.00, 62.00] | 54.00 [48.00, 61.00] | 0.315 |
| History of HBV infection | 62 (80.5) | 40 (85.1) | 0.684 |
| history of cholelithiasis | 15 (19.5) | 4 (8.5) | 0.165 |
| Cirrhosis | 28 (36.4) | 15 (31.9) | 0.756 |
| AFP (>20ng/ml) | 14 (18.2) | 6 (12.8) | 0.587 |
| CA19-9 (>1000U/ml) | 8 (10.4) | 10 (21.3) | 0.159 |
| CEA (>2.5ng/ml) | 48 (62.3) | 18 (38.3) | **0.016** |
| MR Radiographic Characteristics |  |  |  |
| Arterial enhancement patterns |  |  | 0.63 |
| Peripheral rim enhancement | 31 (40.3) | 17 (36.2) |  |
| Diffuse hyperenhancement | 17 (22.1) | 14 (29.8) |  |
| Diffuse hypoenhancement | 29 (37.7) | 16 (34.0) |  |
| Enhancement pattern |  |  | 0.295 |
| Wash-out pattern | 13 (16.9) | 6 (12.8) |  |
| Persistent enhancement | 10 (13.0) | 9 (19.1) |  |
| Gradual enhancement | 49 (63.6) | 25 (53.2) |  |
| No or minimal enhancement | 5 (6.5) | 7 (14.9) |  |
| Irregular tumor margin | 37 (48.1) | 12 (25.5) | **0.021** |
| Peritumoral enhancement | 17 (22.1) | 14 (29.8) | 0.454 |
| Peritumoral biliary dilatation | 29 (37.7) | 21 (44.7) | 0.559 |
| Target sign on DWI | 41 (53.2) | 24 (51.1) | 0.959 |
| Multifocal tumor | 18 (23.4) | 9 (9.1) | 0.742 |
| Tumor diameter(cm) | 46.00 [33.00, 60.00] | 51.00 [39.00, 67.50] | 0.178 |
| Pathologic findings |  |  |  |
| Surgical margin status(R1) | 2 (2.6) | 3 (6.4) | 0.569 |
| Macrovascular invasion | 18 (23.4) | 18 (38.3) | 0.116 |
| Microvascular invasion | 28 (36.4) | 15 (31.9) | 0.756 |
| Histologic differentiation |  |  | 0.99 |
| Well or moderate | 33 (42.9) | 21 (44.7) |  |
| Poor | 44 (57.1) | 26 (55.3) |  |
| Lymph node metastasis | 23 (29.9) | 24 (51.1) | **0.03** |
| T stage |  |  | 0.11 |
| T1a | 32 (41.6) | 12 (25.5) |  |
| T1b | 14 (18.2) | 10 (21.3) |  |
| T2 | 31 (40.3) | 23 (48.9) |  |
| T3 | 0 (0.0) | 2 (4.3) |  |
| TNM stage |  |  | 0.05 |
| ⅠA | 28 (36.4) | 9 (19.1) |  |
| ⅠB | 9 (11.7) | 6 (12.8) |  |
| Ⅱ | 17 (22.1) | 7 (14.9) |  |
| Ⅲ | 23 (29.9) | 25 (53.2) |  |
| Extension of hepatectomy |  |  | 0.907 |
| Minor resection | 48 (62.3) | 28 (59.6) |  |
| Major resection | 29(37.7) | 19 (40.4) |  |
| lymphadenectomy | 37 (48.1) | 21 (44.7) | 0.858 |
| Early recurrence | 48 (62.3) | 29 (61.7) | 1 |
| Radiomics signature(high-risk) | 37 (48.1) | 22 (46.8) | 1 |
| Radiomics nomogram(high-risk) | 44 (57.1) | 29 (61.7) | 1 |
| Abbreviations：HBV, hepatitis B virus; DWI, diffusion weighted imaging; AFP, alpha fetoprotein; CEA, carcinoembryonic antigen; CA19-9, carbohydrate antigen 19-9. | | | |

| Additional file 1: Table S8 Univariable and multivariable Cox regression analysis of DFS within the high-risk group defined by the radiomic signature. | | | | | | |
| --- | --- | --- | --- | --- | --- | --- |
| Characteristics | Univariate Analysis | | | Multivariate Analysis | | |
|  | HR | 95%CI | P | HR | 95%CI | P |
| Clinical Characteristics |  |  |  |  |  |  |
| Sex(female) | 0.873 | 0.507-1.505 | 0.626 |  |  |  |
| age (year) | 0.985 | 0.959-1.012 | 0.266 |  |  |  |
| History of HBV infection | 0.773 | 0.388-1.542 | 0.465 |  |  |  |
| History of cholelithiasis | 1.306 | 0.653-2.613 | 0.451 |  |  |  |
| cirrhosis | 1.483 | 0.826-2.661 | 0.187 |  |  |  |
| CA19-9＞1000U/mL | 1.18 | 0.529-2.631 | 0.685 |  |  |  |
| MR Radiographic Characteristics | |  |  |  |  |  |
| Arterial enhancement patterns |  |  |  |  |  |  |
| Peripheral rim enhancement | ref | ref |  |  |  |  |
| Diffuse hyperenhancement | 0.949 | 0.455-1.982 | 0.89 |  |  |  |
| Diffuse hypoenhancement | 1.306 | 0.716-2.383 | 0.384 |  |  |  |
| Enhancement pattern |  |  |  |  |  |  |
| Wash-out pattern | ref | ref |  |  |  |  |
| Persistent enhancement | 1.109 | 0.312-3.949 | 0.873 |  |  |  |
| Gradual enhancement | 1.249 | 0.524-2.979 | 0.616 |  |  |  |
| No or minimal enhancement | 1.367 | 0.471-3.97 | 0.565 |  |  |  |
| Irregular tumor margin | 1.284 | 0.752-2.194 | 0.36 |  |  |  |
| Peritumoral enhancement | 1.103 | 0.619-1.963 | 0.74 |  |  |  |
| Peritumoral biliary dilatation | 1.434 | 0.834-2.465 | 0.192 |  |  |  |
| Target sign on DWI | 0.768 | 0.443-1.33 | 0.346 |  |  |  |
| Multifocal tumor | 2.151 | 1.183-3.913 | 0.012 | 0.796 | 0.309-2.051 | 0.636 |
| Tumor diameter(cm) | 1.003 | 0.992-1.013 | 0.628 |  |  |  |
| Pathologic findings |  |  |  |  |  |  |
| Surgical margin status (R1) | 5.206 | 1.542-17.574 | 0.008 | 2.069 | 0.498-8.605 | 0.317 |
| Macrovascular invasion | 1.894 | 1.06-3.382 | 0.031 | 0.972 | 0.314-3.008 | 0.96 |
| Microvascular invasion | 2.438 | 1.358-4.377 | 0.003 | 1.601 | 0.770-3.327 | 0.207 |
| Poor differentiation | 1.1 | 0.622-1.944 | 0.743 |  |  |  |
| Lymph node metastasis | 1.083 | 0.625-1.876 | 0.775 |  |  |  |
| T stage |  |  |  |  |  |  |
| T1a | Ref |  |  |  |  |  |
| T1b | 0.837 | 0.386-1.817 | 0.653 | 0.995 | 0.454-2.180 | 0.99 |
| T2 | 2.358 | 1.187-4.682 | 0.014 | 3.320 | 0.864-12.757 | 0.081 |
| T3 | 0.793 | 0.178-3.54 | 0.761 | 1.678 | 0.334-8.430 | 0.529 |
| Adjuvant therapy | 0.521 | 0.288-0.941 | 0.031 | 0.431 | 0.209-0.887 | **0.022** |
| Variables with a p value <0.05 identified on univariable analysis were selected for the multivariable analysis. Abbreviations: DFS, disease-free survival; HR, hazard ratio; 95%CI, 95% confidence intervals. HBV, hepatitis B virus; DWI, diffusion weighted imaging; CA19-9, carbohydrate antigen 19-9，TACE，transhepatic arterial chemotherapy and embolization. | | | | | | |

| Additional file 1: Table S9 Univariable and multivariable Cox regression analysis of overall survival within the high-risk group defined by the radiomic signature. | | | | | | |
| --- | --- | --- | --- | --- | --- | --- |
| Characteristics | Univariate Analysis | | | Multivariate Analysis | | |
|  | HR | 95%CI | P | HR | 95%CI | P |
| Clinical Characteristics |  |  |  |  |  |  |
| Sex(female) | 0.657 | 0.34-1.27 | 0.212 |  |  |  |
| age (year) | 0.996 | 0.966-1.028 | 0.822 |  |  |  |
| History of HBV infection | 1.112 | 0.487-2.538 | 0.801 |  |  |  |
| History of cholelithiasis | 0.592 | 0.208-1.681 | 0.325 |  |  |  |
| cirrhosis | 1.928 | 0.971-3.827 | 0.061 |  |  |  |
| CA19-9＞1000U/mL | 1.363 | 0.527-3.526 | 0.524 |  |  |  |
| MR Radiographic Characteristics | |  |  |  |  |  |
| Arterial enhancement patterns |  |  |  |  |  |  |
| Peripheral rim enhancement | ref | ref |  |  |  |  |
| Diffuse hyperenhancement | 0.973 | 0.402-2.358 | 0.952 |  |  |  |
| Diffuse hypoenhancement | 1.745 | 0.837-3.636 | 0.137 |  |  |  |
| Enhancement pattern |  |  |  |  |  |  |
| Wash-out pattern | ref | ref |  |  |  |  |
| Persistent enhancement | 0.854 | 0.142-5.116 | 0.862 |  |  |  |
| Gradual enhancement | 1.66 | 0.505-5.453 | 0.404 |  |  |  |
| No or minimal enhancement | 0.675 | 0.136-3.361 | 0.632 |  |  |  |
| Irregular tumor margin | 1.254 | 0.655-2.401 | 0.495 |  |  |  |
| Peritumoral enhancement | 1.05 | 0.534-2.065 | 0.888 |  |  |  |
| Peritumoral biliary dilatation | 2.418 | 1.262-4.632 | 0.008 | 2.346 | 1.066-5.163 | **0.034** |
| Target sign on DWI | 0.443 | 0.23-0.853 | 0.015 | 0.396 | 0.193-0.816 | **0.012** |
| Multifocal tumor | 2.157 | 1.072-4.342 | 0.031 | 1.138 | 0.392-3.310 | 0.812 |
| Tumor diameter(cm) | 1.003 | 0.991-1.016 | 0.629 |  |  |  |
| Pathologic findings |  |  |  |  |  |  |
| Surgical margin status (R1) | 4.726 | 1.382-16.158 | 0.013 | 3.008 | 0.780-11.600 | 0.11 |
| Macrovascular invasion | 2.141 | 1.094-4.19 | 0.026 | 0.704 | 0.227-2.186 | 0.544 |
| Microvascular invasion | 1.695 | 0.882-3.257 | 0.113 |  |  |  |
| Poor differentiation | 1.379 | 0.665-2.863 | 0.388 |  |  |  |
| Lymph node metastasis | 2.205 | 1.148-4.235 | 0.018 | 1.322 | 0.621-2.812 | 0.469 |
| T stage |  |  |  |  |  |  |
| T1a |  |  |  |  |  |  |
| T1b | 1.624 | 0.553-4.769 | 0.377 | 1.170 | 0.384-3.566 | 0.782 |
| T2 | 3.851 | 1.424-10.413 | 0.008 | 2.573 | 0.516-12.832 | 0.249 |
| T3 | 1.998 | 0.229-17.422 | 0.531 | 1.033 | 0.103-10.329 | 0.978 |
| Adjuvant therapy | 0.544 | 0.267-1.106 | 0.093 |  |  |  |
| TACE | 0.506 | 0.155-1.652 | 0.259 |  |  |  |
| Ablation therapy | 0.452 | 0.138-1.483 | 0.19 |  |  |  |
| Variables with a p value <0.05 identified on univariable analysis were selected for the multivariable analysis. Abbreviations: HR, hazard ratio; 95%CI, 95% confidence intervals. HBV, hepatitis B virus; DWI, diffusion weighted imaging; CA19-9, carbohydrate antigen 19-9，TACE，transhepatic arterial chemotherapy and embolization. | | | | | | |

| Additional file 1: Table S10 Univariable and multivariable Cox regression analysis of risk factors of DFS within the low-risk group defined by radiomic signature. | | | | | | |
| --- | --- | --- | --- | --- | --- | --- |
| Characteristics | Univariate Analysis | | | Multivariate Analysis | | |
|  | HR | 95%CI | P | HR | 95%CI | P |
| Clinical Characteristics |  |  |  |  |  |  |
| Sex(female) | 1.292 | 0.677-2.466 | 0.437 |  |  |  |
| age (year) | 0.978 | 0.945-1.012 | 0.195 |  |  |  |
| History of HBV infection | 1.513 | 0.589-3.881 | 0.389 |  |  |  |
| History of cholelithiasis | 0.685 | 0.267-1.754 | 0.43 |  |  |  |
| cirrhosis | 0.869 | 0.46-1.643 | 0.666 |  |  |  |
| CA19-9＞1000U/mL | 1.624 | 0.749-3.522 | 0.22 |  |  |  |
| MR Radiographic Characteristics | |  |  |  |  |  |
| Arterial enhancement patterns |  |  |  |  |  |  |
| Peripheral rim enhancement | ref | ref |  |  |  |  |
| Diffuse hyperenhancement | 1.203 | 0.555-2.606 | 0.64 |  |  |  |
| Diffuse hypoenhancement | 1.338 | 0.652-2.749 | 0.428 |  |  |  |
| Enhancement pattern |  |  |  |  |  |  |
| Wash-out pattern | ref | ref |  |  |  |  |
| Persistent enhancement | 2.159 | 0.746-6.245 | 0.156 | 2.119 | 0.730-6.146 | 0.167 |
| Gradual enhancement | 2.022 | 0.758-5.392 | 0.16 | 1.810 | 0.67-4.891 | 0.242 |
| No or minimal enhancement | 11.737 | 2.625-52.481 | 0.001 | 8.597 | 1.861-39.705 | **0.006** |
| Irregular tumor margin | 1.588 | 0.839-3.008 | 0.156 |  |  |  |
| Peritumoral enhancement | 1.348 | 0.643-2.828 | 0.429 |  |  |  |
| Peritumoral biliary dilatation | 2.121 | 1.124-4.005 | 0.02 | 1.889 | 0.974-3.663 | 0.06 |
| Target sign on DWI | 0.862 | 0.469-1.586 | 0.633 |  |  |  |
| Multifocal tumor | 1.501 | 0.686-3.285 | 0.309 |  |  |  |
| Tumor diameter(cm) | 0.998 | 0.98-1.016 | 0.816 |  |  |  |
| Pathologic findings |  |  |  |  |  |  |
| Surgical margin status (R1) | 0.734 | 0.1-5.372 | 0.761 |  |  |  |
| Macrovascular invasion | 1.337 | 0.67-2.669 | 0.411 |  |  |  |
| Microvascular invasion | 1.677 | 0.897-3.136 | 0.105 |  |  |  |
| Poor differentiation | 1.439 | 0.782-2.647 | 0.242 |  |  |  |
| Lymph node metastasis | 1.59 | 0.742-3.406 | 0.233 |  |  |  |
| T stage |  |  |  |  |  |  |
| T1a | Ref |  |  |  |  |  |
| T1b | 0.909 | 0.299-2.761 | 0.867 |  |  |  |
| T2 | 1.686 | 0.764-3.719 | 0.196 |  |  |  |
| T3 | --- | --- | --- |  |  |  |
| Adjuvant therapy | 1.371 | 0.736-2.552 | 0.32 |  |  |  |
| Variables with a p value <0.05 identified on univariable analysis were selected for the multivariable analysis. Abbreviations: DFS, disease-free survival; HR, hazard ratio; 95%CI, 95% confidence intervals. HBV, hepatitis B virus; DWI, diffusion weighted imaging; CA19-9, carbohydrate antigen 19-9，TACE，transhepatic arterial chemotherapy and embolization. | | | | | | |

| Additional file 1: Table S11 Univariable and multivariable Cox regression analysis of overall survival within the low-risk group defined by the radiomic signature. | | | | | | |
| --- | --- | --- | --- | --- | --- | --- |
| Characteristics | Univariate Analysis | | | Multivariate Analysis | | |
|  | HR | 95%CI | P | HR | 95%CI | P |
| Clinical Characteristics |  |  |  |  |  |  |
| Sex(female) | 1.879 | 0.848-4.164 | 0.12 |  |  |  |
| age (year) | 0.993 | 0.953-1.034 | 0.72 |  |  |  |
| History of HBV infection | 1.022 | 0.3-3.486 | 0.972 |  |  |  |
| History of cholelithiasis | 0.787 | 0.235-2.632 | 0.697 |  |  |  |
| cirrhosis | 0.629 | 0.264-1.497 | 0.294 |  |  |  |
| CA19-9＞1000U/mL | 2.225 | 0.88-5.627 | 0.091 |  |  |  |
| MR Radiographic Characteristics | |  |  |  |  |  |
| Arterial enhancement patterns |  |  |  |  |  |  |
| Peripheral rim enhancement | ref | ref |  |  |  |  |
| Diffuse hyperenhancement | 0.981 | 0.349-2.761 | 0.971 |  |  |  |
| Diffuse hypoenhancement | 1.436 | 0.594-3.471 | 0.421 |  |  |  |
| Enhancement pattern |  |  |  |  |  |  |
| Wash-out pattern | ref | ref |  |  |  |  |
| Persistent enhancement | 2.27 | 0.586-8.797 | 0.236 | 1.968 | 0.505-7.670 | 0.329 |
| Gradual enhancement | 1.827 | 0.52-6.419 | 0.347 | 1.092 | 0.286-4.168 | 0.898 |
| No or minimal enhancement | 18.322 | 3.402-98.671 | 0.001 | 7.936 | 1.362-46.237 | **0.021** |
| Irregular tumor margin | 2.824 | 1.27-6.282 | 0.011 | 2.187 | 0.894-5.349 | 0.086 |
| Peritumoral enhancement | 1.202 | 0.411-3.517 | 0.737 |  |  |  |
| Peritumoral biliary dilatation | 3.569 | 1.546-8.236 | 0.003 | 2.315 | 0.909-5.893 | 0.078 |
| Target sign on DWI | 0.875 | 0.403-1.896 | 0.735 |  |  |  |
| Multifocal tumor | 1.498 | 0.6-3.738 | 0.387 |  |  |  |
| Tumor diameter(cm) | 0.995 | 0.972-1.018 | 0.659 |  |  |  |
| Pathologic findings |  |  |  |  |  |  |
| Surgical margin status (R1) | 1.926 | 0.251-14.755 | 0.528 |  |  |  |
| Macrovascular invasion | 1.643 | 0.711-3.796 | 0.246 |  |  |  |
| Microvascular invasion | 1.335 | 0.59-3.02 | 0.488 |  |  |  |
| Poor differentiation | 1.455 | 0.673-3.146 | 0.34 |  |  |  |
| Lymph node metastasis | 2.331 | 1.061-5.12 | 0.035 | 2.499 | 1.030-6.063 | **0.043** |
| T stage |  |  |  |  |  |  |
| T1a |  |  |  |  |  |  |
| T1b | 0.988 | 0.216-4.528 | 0.988 |  |  |  |
| T2 | 1.902 | 0.841-4.301 | 0.122 |  |  |  |
| T3 |  | NA |  |  |  |  |
| Adjuvant therapy | 0.906 | 0.387-2.12 | 0.819 |  |  |  |
| TACE | 0 | 0-Inf | 0.998 |  |  |  |
| Ablation therapy | 0.398 | 0.054-2.959 | 0.368 |  |  |  |
| Variables with a p value <0.05 identified on univariable analysis were selected for the multivariable analysis. Abbreviations: HR, hazard ratio; 95%CI, 95% confidence intervals. HBV, hepatitis B virus; DWI, diffusion weighted imaging; CA19-9, carbohydrate antigen 19-9，TACE，transhepatic arterial chemotherapy and embolization. | | | | | | |
| Additional file 1: Table S12Univariable and multivariable Cox regression analysis of DFS within the high-risk group defined by the radiomic nomogram. | | | | | | |
| Characteristics | Univariate Analysis | | | Multivariate Analysis | | |
|  | HR | 95%CI | P | HR | 95%CI | P |
| Clinical Characteristics |  |  |  |  |  |  |
| Sex(female) | 0.925 | 0.568-1.506 | 0.755 |  |  |  |
| age (year) | 0.984 | 0.96-1.008 | 0.19 |  |  |  |
| History of HBV infection | 1.305 | 0.665-2.564 | 0.439 |  |  |  |
| History of cholelithiasis | 1.312 | 0.699-2.462 | 0.399 |  |  |  |
| cirrhosis | 1.302 | 0.781-2.17 | 0.311 |  |  |  |
| CA19-9＞1000U/mL | 1.522 | 0.807-2.871 | 0.195 |  |  |  |
| MR Radiographic Characteristics | | |  |  |  |  |
| Arterial enhancement patterns |  |  |  |  |  |  |
| Peripheral rim enhancement | ref | ref |  |  |  |  |
| Diffuse hyperenhancement | 1.006 | 0.529-1.913 | 0.985 |  |  |  |
| Diffuse hypoenhancement | 1.611 | 0.924-2.811 | 0.093 |  |  |  |
| Enhancement pattern |  |  |  |  |  |  |
| Wash-out pattern | ref | ref |  |  |  |  |
| Persistent enhancement | 0.967 | 0.359-2.606 | 0.948 |  |  |  |
| Gradual enhancement | 1.411 | 0.683-2.915 | 0.352 |  |  |  |
| No or minimal enhancement | 1.464 | 0.594-3.612 | 0.408 |  |  |  |
| Irregular tumor margin | 1.318 | 0.811-2.141 | 0.266 |  |  |  |
| Peritumoral enhancement | 1.154 | 0.676-1.97 | 0.6 |  |  |  |
| Peritumoral biliary dilatation | 1.23 | 0.757-1.997 | 0.403 |  |  |  |
| Target sign on DWI | 0.667 | 0.409-1.09 | 0.106 |  |  |  |
| Multifocal tumor | 1.592 | 0.935-2.711 | 0.087 |  |  |  |
| Tumor diameter(cm) | 1 | 0.991-1.01 | 0.948 |  |  |  |
| Pathologic findings |  |  |  |  |  |  |
| Surgical margin status (R1) | 4.533 | 1.376-14.936 | 0.013 | 3.826 | 1.153-12.694 | **0.028** |
| Macrovascular invasion | 1.288 | 0.775-2.141 | 0.329 |  |  |  |
| Microvascular invasion | 1.282 | 0.787-2.088 | 0.318 |  |  |  |
| Poor differentiation | 1.036 | 0.616-1.743 | 0.893 |  |  |  |
| Lymph node metastasis | 1.374 | 0.838-2.254 | 0.208 |  |  |  |
| T stage |  |  |  |  |  |  |
| T1a | Ref |  |  |  |  |  |
| T1b | 0.687 | 0.328-1.442 | 0.321 |  |  |  |
| T2 | 1.274 | 0.716-2.268 | 0.41 |  |  |  |
| T3 | 0.554 | 0.127-2.418 | 0.432 |  |  |  |
| Adjuvant therapy | 0.569 | 0.339-0.953 | 0.032 | 0.592 | 0.351-0.998 | **0.049** |
| Variables with a p value <0.05 identified on univariable analysis were selected for the multivariable analysis. Abbreviations: DFS, disease-free survival; HR, hazard ratio; 95%CI, 95% confidence intervals. HBV, hepatitis B virus; DWI, diffusion weighted imaging; CA19-9, carbohydrate antigen 19-9，TACE，transhepatic arterial chemotherapy and embolization. | | | | | | |

| Additional file 1: Table S13 Univariable and multivariable Cox regression analysis of overall survival within the high-risk group defined by the radiomic nomogram. | | | | | | |
| --- | --- | --- | --- | --- | --- | --- |
| Characteristics | Univariate Analysis | | | Multivariate Analysis | | |
|  | HR | 95%CI | P | HR | 95%CI | P |
| Clinical Characteristics |  |  |  |  |  |  |
| Sex(female) | 0.823 | 0.452-1.497 | 0.523 |  |  |  |
| age (year) | 0.993 | 0.965-1.022 | 0.629 |  |  |  |
| History of HBV infection | 1.282 | 0.571-2.882 | 0.547 |  |  |  |
| History of cholelithiasis | 0.719 | 0.282-1.835 | 0.491 |  |  |  |
| cirrhosis | 1.34 | 0.715-2.509 | 0.361 |  |  |  |
| CA19-9＞1000U/mL | 1.585 | 0.758-3.312 | 0.221 |  |  |  |
| MR Radiographic Characteristics | | |  |  |  |  |
| Arterial enhancement patterns |  |  |  |  |  |  |
| Peripheral rim enhancement | ref | ref |  |  |  |  |
| Diffuse hyperenhancement | 0.762 | 0.326-1.783 | 0.53 |  |  |  |
| Diffuse hypoenhancement | 1.921 | 0.993-3.715 | 0.053 |  |  |  |
| Enhancement pattern |  |  |  |  |  |  |
| Wash-out pattern | ref | ref |  |  |  |  |
| Persistent enhancement | 0.886 | 0.198-3.973 | 0.874 |  |  |  |
| Gradual enhancement | 2.054 | 0.724-5.824 | 0.176 |  |  |  |
| No or minimal enhancement | 1.141 | 0.304-4.275 | 0.845 |  |  |  |
| Irregular tumor margin | 1.483 | 0.816-2.692 | 0.196 |  |  |  |
| Peritumoral enhancement | 1.126 | 0.596-2.129 | 0.714 |  |  |  |
| Peritumoral biliary dilatation | 2.183 | 1.195-3.988 | 0.011 | 2.622 | 1.371-5.014 | **0.004** |
| Target sign on DWI | 0.479 | 0.262-0.876 | 0.017 | 0.498 | 0.268-0.924 | **0.027** |
| Multifocal tumor | 1.626 | 0.85-3.112 | 0.142 |  |  |  |
| Tumor diameter(cm) | 1 | 0.988-1.011 | 0.986 |  |  |  |
| Pathologic findings |  |  |  |  |  |  |
| Surgical margin status (R1) | 5.298 | 1.563-17.965 | 0.007 | 3.281 | 0.891-12.081 | 0.074 |
| Macrovascular invasion | 1.556 | 0.849-2.852 | 0.152 |  |  |  |
| Microvascular invasion | 1.151 | 0.633-2.095 | 0.644 |  |  |  |
| Poor differentiation | 1.453 | 0.734-2.879 | 0.284 |  |  |  |
| Lymph node metastasis | 2.092 | 1.154-3.793 | 0.015 | 2.377 | 1.249-4.522 | **0.008** |
| T stage |  |  |  |  |  |  |
| T1a |  |  |  |  |  |  |
| T1b | 1.077 | 0.427-2.72 | 0.875 |  |  |  |
| T2 | 1.812 | 0.837-3.926 | 0.132 |  |  |  |
| T3 | 1.269 | 0.159-10.142 | 0.822 |  |  |  |
| Adjuvant therapy | 0.475 | 0.247-0.915 | 0.026 | 0.291 | 0.143-0.591 | **0.001** |
| TACE | 0.572 | 0.138-2.365 | 0.44 |  |  |  |
| Ablation therapy | 0.164 | 0.023-1.192 | 0.074 |  |  |  |
| Variables with a p value <0.05identified on univariable analysis were selected for the multivariable analysis. Abbreviations: HR, hazard ratio; 95%CI, 95% confidence intervals. HBV, hepatitis B virus; DWI, diffusion weighted imaging; CA19-9, carbohydrate antigen 19-9，TACE，transhepatic arterial chemotherapy and embolization. | | | | | | |

| Additional file 1: Table S14 Univariable and multivariable Cox regression analysis of risk factors of DFS within the low-risk group defined by the radiomic nomogram | | | | | | |
| --- | --- | --- | --- | --- | --- | --- |
| Characteristics | Univariate Analysis | | | Multivariate Analysis | | |
|  | HR | 95%CI | P | HR | 95%CI | P |
| Clinical Characteristics |  |  |  |  |  |  |
| Sex(female) | 1.094 | 0.497-2.41 | 0.823 |  |  |  |
| age (year) | 0.986 | 0.947-1.025 | 0.468 |  |  |  |
| History of HBV infection | 0.789 | 0.297-2.095 | 0.634 |  |  |  |
| History of cholelithiasis | 0.498 | 0.15-1.657 | 0.256 |  |  |  |
| cirrhosis | 0.701 | 0.319-1.544 | 0.378 |  |  |  |
| CA19-9＞1000U/mL | 0.856 | 0.259-2.832 | 0.799 |  |  |  |
| MR Radiographic Characteristics | | |  |  |  |  |
| Arterial enhancement patterns |  |  |  |  |  |  |
| Peripheral rim enhancement | ref | ref |  |  |  |  |
| Diffuse hyperenhancement | 1.197 | 0.472-3.038 | 0.705 |  |  |  |
| Diffuse hypoenhancement | 1.276 | 0.55-2.963 | 0.57 |  |  |  |
| Enhancement pattern |  |  |  |  |  |  |
| Wash-out pattern | ref | ref |  |  |  |  |
| Persistent enhancement | 4.129 | 0.872-19.55 | 0.074 |  |  |  |
| Gradual enhancement | 3.85 | 0.88-16.85 | 0.073 |  |  |  |
| No or minimal enhancement | NA |  |  |  |  |  |
| Irregular tumor margin | 1.391 | 0.633-3.054 | 0.411 |  |  |  |
| Peritumoral enhancement | 1.458 | 0.623-3.411 | 0.384 |  |  |  |
| Peritumoral biliary dilatation | 1.986 | 0.92-4.284 | 0.08 |  |  |  |
| Target sign on DWI | 1.215 | 0.591-2.496 | 0.597 |  |  |  |
| Multifocal tumor | 1.829 | 0.685-4.886 | 0.228 |  |  |  |
| Tumor diameter(cm) | 1.006 | 0.986-1.026 | 0.58 |  |  |  |
| Pathologic findings |  |  |  |  |  |  |
| Surgical margin status (R1) | 1.139 | 0.152-8.52 | 0.899 |  |  |  |
| Macrovascular invasion | 1.386 | 0.563-3.411 | 0.478 |  |  |  |
| Microvascular invasion | 0.861 | 0.299-2.475 | 0.78 |  |  |  |
| Poor differentiation | 1.069 | 0.509-2.244 | 0.86 |  |  |  |
| Lymph node metastasis | 1.59 | 0.742-3.406 | 0.233 |  |  |  |
| T stage |  |  |  |  |  |  |
| T1a | Ref |  |  |  |  |  |
| T1b | 0.909 | 0.299-2.761 | 0.867 |  |  |  |
| T2 | 1.686 | 0.764-3.719 | 0.196 |  |  |  |
| T3 | --- | --- | --- |  |  |  |
| Adjuvant therapy | 1.346 | 0.638-2.838 | 0.435 |  |  |  |
| Variables with a p value <0.05 identified on univariable analysis were selected for the multivariable analysis. Abbreviations: DFS, disease-free survival; HR, hazard ratio; 95%CI, 95% confidence intervals. HBV, hepatitis B virus; DWI, diffusion weighted imaging; CA19-9, carbohydrate antigen 19-9，TACE，transhepatic arterial chemotherapy and embolization. | | | | | | |

| Additional file 1: Table S15 Univariable and multivariable Cox regression analysis of overall survival within the low-risk group defined by the radiomic nomogram. | | | | | | |
| --- | --- | --- | --- | --- | --- | --- |
| Characteristics | Univariate Analysis | | | Multivariate Analysis | | |
|  | HR | 95%CI | P | HR | 95%CI | P |
| Clinical Characteristics |  |  |  |  |  |  |
| Sex(female) | 1.714 | 0.65-4.518 | 0.276 |  |  |  |
| age (year) | 1.005 | 0.958-1.054 | 0.848 |  |  |  |
| History of HBV infection | 0.578 | 0.158-2.111 | 0.407 |  |  |  |
| History of cholelithiasis | 0.662 | 0.152-2.874 | 0.581 |  |  |  |
| cirrhosis | 0.654 | 0.234-1.823 | 0.417 |  |  |  |
| CA19-9＞1000U/mL | 1.182 | 0.271-5.153 | 0.824 |  |  |  |
| MR Radiographic Characteristics | | |  |  |  |  |
| Arterial enhancement patterns |  |  |  |  |  |  |
| Peripheral rim enhancement | ref | ref |  |  |  |  |
| Diffuse hyperenhancement | 1.462 | 0.47-4.555 | 0.512 |  |  |  |
| Diffuse hypoenhancement | 1.248 | 0.419-3.717 | 0.691 |  |  |  |
| Enhancement pattern |  |  |  |  |  |  |
| Wash-out pattern | ref | ref |  |  |  |  |
| Persistent enhancement | 3.317 | 0.664-16.571 | 0.144 |  |  |  |
| Gradual enhancement | 2.086 | 0.453-9.613- | 0.346 |  |  |  |
| No or minimal enhancement | NA |  |  |  |  |  |
| Irregular tumor margin | 1.898 | 0.711-5.066 | 0.201 |  |  |  |
| Peritumoral enhancement | 1.138 | 0.327-3.959 | 0.839 |  |  |  |
| Peritumoral biliary dilatation | 3.844 | 1.394-10.604 | 0.009 | 2.398 | 0.827--6.96 | 0.107 |
| Target sign on DWI | 0.981 | 0.397-2.429 | 0.968 |  |  |  |
| Multifocal tumor | 2.179 | 0.78-6.082 | 0.137 |  |  |  |
| Tumor diameter(cm) | 1.007 | 0.983-1.033 | 0.562 |  |  |  |
| Pathologic findings |  |  |  |  |  |  |
| Surgical margin status (R1) | 3.067 | 0.377-24.979 | 0.295 |  |  |  |
| Macrovascular invasion | 1.852 | 0.665-5.156 | 0.238 |  |  |  |
| Microvascular invasion | 0.659 | 0.151-2.872 | 0.578 |  |  |  |
| Poor differentiation | 1.026 | 0.403-2.613 | 0.957 |  |  |  |
| Lymph node metastasis | 3.34 | 1.295-8.611 | 0.013 | 2.422 | 0.826-7.096 | 0.107 |
| T stage |  |  |  |  |  |  |
| T1a |  |  |  |  |  |  |
| T1b | 1.806 | 0.451-7.239 | 0.404 | 1.852 | 0.443-7.744 | 0.399 |
| T2 | 3 | 1.089-8.264 | 0.034 | 1.804 | 0.605-5.378 | 0.29 |
| T3 |  | NA |  |  | NA |  |
| Adjuvant therapy | 1.29 | 0.475-3.506 | 0.618 |  |  |  |
| TACE | 0.543 | 0.072-4.121 | 0.555 |  |  |  |
| Ablation therapy | 1.226 | 0.349-4.304 | 0.75 |  |  |  |
| Variables with a p value <0.05 identified on univariable analysis were selected for the multivariable analysis. Abbreviations: HR, hazard ratio; 95%CI, 95% confidence intervals. HBV, hepatitis B virus; DWI, diffusion weighted imaging; CA19-9, carbohydrate antigen 19-9，TACE，transhepatic arterial chemotherapy and embolization. | | | | | | |
